# Combining newborn metabolic and DNA analysis for second-tier testing of methylmalonic acidemia

**DOI:** 10.1038/s41436-018-0272-5

**Published:** 2018-09-13

**Authors:** Gang Peng, Peidong Shen, Neeru Gandotra, Anthony Le, Eula Fung, Laura Jelliffe-Pawlowski, Ronald W. Davis, Gregory M. Enns, Hongyu Zhao, Tina M. Cowan, Curt Scharfe

**Affiliations:** 1 Department of Genetics, Yale University School of Medicine, New Haven, CT USA; 2 Department of Biostatistics, Yale University School of Public Health, New Haven, CT USA; 3 Stanford Genome Technology Center, Stanford University, Palo Alto, CA USA; 4 Department of Pathology, Stanford University School of Medicine, Stanford, CA USA; 5 Department of Epidemiology and Biostatistics, University of California San Francisco School of Medicine, San Francisco, CA USA; 6 Department of Pediatrics, Stanford University School of Medicine, Stanford, CA USA

**Keywords:** newborn screening, inborn metabolic disorders, DNA diagnostics, next-generation sequencing, machine learning

## Abstract

**Purpose:**

Improved second-tier tools are needed to reduce false-positive outcomes in newborn screening (NBS) for inborn metabolic disorders on the Recommended Universal Screening Panel (RUSP).

**Methods:**

We designed an assay for multiplex sequencing of 72 metabolic genes (RUSPseq) from newborn dried blood spots. Analytical and clinical performance was evaluated in 60 screen-positive newborns for methylmalonic acidemia (MMA) reported by the California Department of Public Health NBS program. Additionally, we trained a Random Forest machine learning classifier on NBS data to improve prediction of true and false-positive MMA cases.

**Results:**

Of 28 MMA patients sequenced, we found two pathogenic or likely pathogenic (P/LP) variants in a MMA-related gene in 24 patients, and one pathogenic variant and a variant of unknown significance (VUS) in 1 patient. No such variant combinations were detected in MMA false positives and healthy controls. Random Forest–based analysis of the entire NBS metabolic profile correctly identified the MMA patients and reduced MMA false-positive cases by 51%. MMA screen-positive newborns were more likely of Hispanic ethnicity.

**Conclusion:**

Our two-pronged approach reduced false positives by half and provided a reportable molecular finding for 89% of MMA patients. Challenges remain in newborn metabolic screening and DNA variant interpretation in diverse multiethnic populations.

## Introduction

Newborn screening (NBS) using tandem mass spectrometry (MS/MS) represents a major advance in our ability to detect inborn metabolic disorders that have historically caused significant morbidity and mortality in children.^1–3^ Using MS/MS, more than 40 metabolic disorders on the Recommended Universal Screening Panel (RUSP) can now be detected from newborn dried blood spots (DBS), the common specimen collected by heel stick shortly after birth.^4,5^ While beneficial in most respects, MS/MS screening is tuned to maximize the number of newborns identified, with sensitivity favored over specificity. This approach increases the number of false-positive results, leading to considerable emotional and financial burdens of follow-up testing, unneeded medical precautions for false-positive cases, and diagnostic delays for some infants.^6^ To reduce the number of false-positive cases without compromising sensitivity, screen-positive results are followed by second-tier testing at higher specificity.^7^ As such, second-tier tests measure more specific disease markers (e.g., organic acids) to confirm (true positive) or reject (false positive) the primary screen result. Second-tier tests are typically not part of the primary screen due to assay complexity, limited throughput, analysis time, and cost.^7,8^ However, both primary and secondary screening utilizes the original newborn DBS to avoid a new blood draw and minimize turnaround time.

The advent of rapid, inexpensive next-generation sequencing (NGS) promises to revolutionize newborn screening.^5,9^ Incorporating NGS-based analysis at the earliest stage in the screening process could drastically streamline the diagnostic work-up following an abnormal NBS result, but has several challenges. Newborn DBS samples contain only small and varying amounts of blood, from which multiple punches are taken for NBS for the various conditions on the panel. The small amount of dried blood remaining limits the amount of extractable DNA for use in second-tier testing. Previous studies using residual DBS for NGS either required large amounts of DBS material, or used whole-genome amplification for sequence library preparation.^10–13^ The feasibility of exome and genome sequencing from two 3-mm DBS punches without whole-genome amplification was recently described.^14^ Despite dramatic reductions in sequencing costs, exome sequencing still is relatively expensive compared with the cost of NBS ($15–$150 depending on the state), which is often covered by health insurance.^15^ A less expensive and more efficient approach is multiplex gene sequencing from DBS, using a panel of genes relevant to the specific NBS condition or biochemical profile detected in the primary MS/MS screen.

Here, we adapted a validated multiplex NGS technology^16^ for sequencing of 72 genes for inborn metabolic disorders (RUSPseq) from a single 3-mm DBS punch, and used it to evaluate archived DBS from newborns that screened positive for methylmalonic acidemia (MMA) by the California Department of Public Health NBS program. MMA screening is fraught with false-positive cases that require second-tier confirmation using liquid chromatography –tandem mass spectrometry (LC-MS/MS),^17^ while DNA testing is necessary to reach a final diagnosis and to identify which of several genes is responsible and the severity of the specific variant.^18^ To further improve MMA screening using MS/MS, we also developed a statistical approach using machine learning that significantly reduced the number of false-positive MMA cases. In addition to the two primary MS/MS analytes currently used in MMA screening, our novel method utilizes information on the entire MS/MS metabolic profile measured at birth.

## Materials and methods

### Study specimens and NBS data

This study was approved by the Institutional Review Boards at Yale University (protocol ID 1505015917), Stanford University (protocol ID 30618) and the State of California Committee for the Protection of Human Subjects (protocol ID 13-05-1236). De-identified residual DBS samples from 80 newborns from the California Biobank Program were used to validate the RUSPseq assay. These samples included 30 confirmed MMA cases, 30 MMA screen false positives, and 20 DBS from healthy controls (Supplementary Table 1). In addition, we evaluated metabolic data from a larger cohort of 803 newborns, consisting of 103 cases with confirmed MMA (24 mut^0^, 26 mut^-^; 45 CblC, D or F; 3 CblA or B; and 5 unclassified MMA), 502 screen false positives, and 198 healthy controls. All newborns had routine MS/MS metabolic screening performed through the California NBS program between 2005 and 2015. The 56 MS/MS analytes included free carnitine, acylcarnitines, amino acids, and calculated ratios. Additional data collected included newborn race/ethnicity, gestational age (GA, in days), birth weight (in grams), total parenteral nutrition (yes or no), and newborn age at blood collection (in hours).

### NBS metabolic data analysis

We performed a retrospective analysis of NBS data from 803 newborns that focused on 46 of the 56 MS/MS analytes. Ten of the 56 analytes had missing data in more than 15% of the samples, and were removed from the analysis. If analytes had missing data in 15% or less of the samples, analyte median values were used to impute missing data. We first compared analyte levels between MMA true positives, false positives, and controls (Supplementary Figure 1). Analysis of variance (ANOVA) was used to compare the 46 analytes between three specific phenotypic subgroups of 95 MMA patients (24 mut^0^, 26 mut^-^, and 45 CblC, D or F). The 3 patients with CblA or B and 5 patients with unclassified MMA were removed from analysis due to small sample size. As differences in gestational age (GA) may be associated with distinct metabolic profiles,^19,20^ we further stratified newborns into two subgroups, 193 preterm (GA ≤ 37 weeks) and 501 full-term (GA > 37 weeks). Of 803 newborns, 109 had no GA information available and were removed from analysis. While NBS programs collect DBS from virtually every newborn, additional outcome data such as GA are not always provided by the referring hospitals. In the second analysis we studied the newborn metabolic patterns of 46 analytes using Random Forest (RF).^21^ We divided the 605 MMA screen positives (103 true positive, 502 false positive) into ten sample groups with stratification of approximately equal size and used a tenfold cross validation to assess the performance of RF. At each validation step, nine sample groups were combined for training, while one group of blinded samples was used for testing. In result of the cross validation, RF classified each of the 605 samples as either a MMA true or false positive. Only RF assignments from testing samples (and not from training) were used to plot the receiver operating characteristic (ROC) curve (Fig. 2a). The synthetic minority oversampling technique (SMOTE)^22^ method was applied to correct for the imbalance in sample size from a larger number of false positives than true positives. The mean decrease in accuracy (MDA) index was used to measure the contribution of individual analytes in the RF model.^23^ MDA analysis was performed for two different RF models with the relative importance of each analyte and covariate ranked from top to bottom. The first RF model included 46 MS/MS analytes (Fig. 2b), while the second model included the 46 analytes and additional covariates of birth weight, total parenteral nutrition, GA, and newborn age at blood collection (Supplementary Figure 2). RF was also applied to predict MMA false positives in the 60 MMA screen-positive sequenced samples (testing set) and using the remaining 545 samples as a training set (Supplementary Table 1). In addition, to study metabolic patterns in MMA phenotypic subgroups, separate RF analyses were performed for 50 mut^0/-^ and 45 CblC, D or F patients, respectively (Supplementary Figure 3). To separate mut^0/-^ from CblC, D or F patients using RF, a tenfold cross validation was performed after dividing the 95 samples into ten sample groups of 5 mut^0/-^ and 4–5 CblC, D or F patients in each group. At each validation step, nine sample groups were combined for training, while one group of blinded samples was used for testing. Only RF assignments from testing samples (and not from training) were used to calculate the error rate for classifying mut^0/-^ from CblC, D or F patients.

### DNA extraction from DBS

A single 3-mm punch was taken from each DBS using a PE Wallac instrument (Perkin Elmer, Santa Clara, CA, USA) and deposited into a 96-well plate. Three blank paper spots were punched between each sample to prevent cross-contamination. DBS punch spots were washed twice with 180 µL of 10 mM NaOH. Each punch spot was then suspended in 50 µL of 10 mM NaOH solution and heated at 99 °C for 15 min in an Applied Biosystems GeneAmp PCR System 9700 (Life Technologies, Grand Island, NY, USA). The supernatant, containing eluted DNA, was mixed by pipetting and then transferred to a clean tube containing 50 µL of 20 mM TrisCL pH 7.5. Two samples (D3, C11 in Supplementary Table 1) of the 80 DBS failed in the DNA extraction.

### RUSPseq design and sequence data analysis

Detailed information for RUSPseq is provided in Supplementary Information. Briefly, target-specific forward and reverse primers were designed for 939 amplicons including all exons and 20 bp of flanking intronic sequence of 72 genes (362,013 bp) based on hg19/GRCh37 (Supplementary Table 2). Establishing single-tube multiplex amplification of 72 genes required primer pool rebalancing, which included increasing or lowering the concentration of specific primers, replacing of failed primers, repeated sequencing, and analysis. Rebalancing minimized amplicon dropout and nonspecific amplification and achieved a 99% target base coverage from <10 ng of DBS DNA extracted from a single DBS punch. We sequenced 78 samples in four Illumina MiSeq runs by multiplexing 17 to 22 samples per run. A no-template water control was included in each run. Following sample de-multiplexing and sequence read alignment (GRCh38 reference assembly), quality control (QC) metrics were extracted for each sample, including total number of reads, percent reads that were properly paired and mapped to the human genome, read depths for each amplicon, and read depth for individual base pairs within the target region (Supplementary Figure 4). DNA variant calling was performed using GATK (version 3.6-0-g89b7209) (ref. ^24^) with parameters as described previously.^16^ ANNOVAR^25^ was used to annotate variants with the corresponding Human Genome Variation Society (HGVS) DNA and protein level nomenclature in combination with public information relevant for variant annotation from OMIM, dbSNP, ClinVar,^26^ and ExAC.^27^ Our sequencing pipeline uses publicly available bioinformatics tools to facilitate the deployability of this workflow in the clinical molecular laboratory. The custom script for data analysis is available at https://github.com/peng-gang/TGPipeline. For each sample (Fig. 3, Supplementary Table 1), sequence variants were classified as pathogenic (P), likely pathogenic (LP), likely benign, benign, or of unknown significance (VUS) based on American College of Medical Genetics and Genomics (ACMG) standards and guidelines for interpretation of sequence variants.^28^

## Results

### Newborn metabolic data analysis

MMA screen-positive cases are identified by C3 acylcarnitine ≥6.5 µmol/L or C3/C2 ratio ≥0.25. Both C3 and C3/C2 are equally important primary NBS analytes.^29^ Between 2005 and 2015, the California NBS program identified 605 MMA screen positives including 103 MMA patients and 502 screen false positives. Notably, 4 of 103 MMA patients had C3 and C3/C2 values below the established thresholds and thus were not technically screen positive for MMA. These 4 cases were screen positive for other NBS metabolic conditions, and elevated MMA levels were seen during follow-up testing, which ultimately lead to their MMA diagnosis. To investigate newborn metabolic patterns, we performed an analysis of 46 NBS metabolic analytes in 605 MMA screen positives and 198 controls (Supplementary Figure 1). Not unexpectedly, we detected significant difference in C3 between MMA patients and controls (*p* = 5.3e–28), and a relatively smaller difference between patients and false positives (*p* = 5.8e–3). Significant differences between MMA patients and false positives were also found for C2 (*p* = 9.1e–14), C3/C2 (*p* = 1.2e–10), and C18:2 (*p* = 5.3e–28), and methionine (*p* = 1.5e–8), arginine/ornithine (*p* = 3.2e–13), and leucine/alanine (*p* = 4.5e–10). Methionine showed significant differences in MMA phenotypic subgroups, with relatively higher methionine levels in patients with mutase deficiencies (mut^0^ or mut^-^) compared with remethylation defects (CblC, D or F), and relatively higher methionine levels in preterm false positives (Supplementary Figure 5). Overall, there were much fewer differences for NBS analytes between preterm newborns with MMA and preterm newborns in the healthy control group, indicating that preterm newborns are metabolically similar.

### Evaluation of newborn race/ethnicity, gestational age, and birth weight

**Fig. 1:**
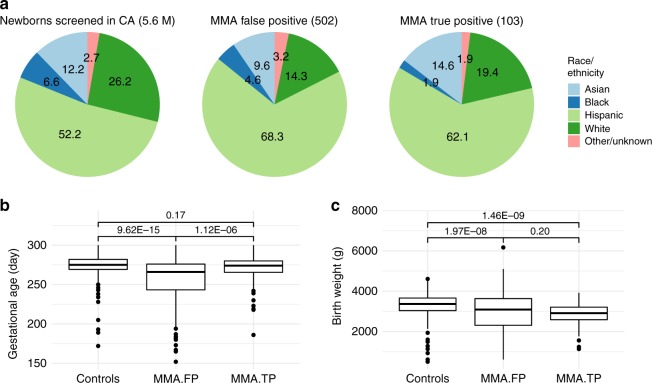
**Newborns screened and methylmalonic acidemia** **(MMA) screen-positive cases by race/ethnicity.** (**a**) Distribution of race/ethnicity of more than 5.6 million healthy newborns, and 502 false-positive MMA cases (MMA.FP) and 103 true-positive MMA patients (MMA.TP) identified in the California newborn screening (NBS) program between 2005 and 2015. (**b**) Gestational age (GA) in days and (**c**) birth weight in grams for MMA screen positive cases and matched healthy controls. The *p* value of a *t* test shows a statistical significant difference between group pairs, with a tendency for MMA.FP to be born premature, and an overall lower birth weight of all MMA screen-positive newborns.

The prevalence of specific disorders detectable through NBS is known to vary widely between racial/ethnic groups.^30^ We assessed the race/ethnicity profile of MMA screen-positive newborns in California’s NBS program (Fig. 1a), and found a significantly higher prevalence of Hispanic newborns among the 605 MMA screen-positive cases than among the 5.6 million newborns screened in California during this same time period of 2005–2015 (*p* = 4.27e–14). Based on recent reports that metabolic profiles may vary by gestational age (GA),^19,20^ we next compared GA between MMA screen-positive cases and healthy newborns (Fig. 1b). Within the group of MMA false positives, there was a significantly higher proportion of preterm (GA ≤37 weeks) compared with full-term (>37 weeks) births. A separate analysis of newborn birth weight (normal: 2500–4000 grams) showed a relatively lower birth weight for MMA screen-positive newborns (both true and false positives) compared with healthy controls (Fig. 1c).

### Newborn metabolic pattern analysis using Random Forest

**Fig. 2:**
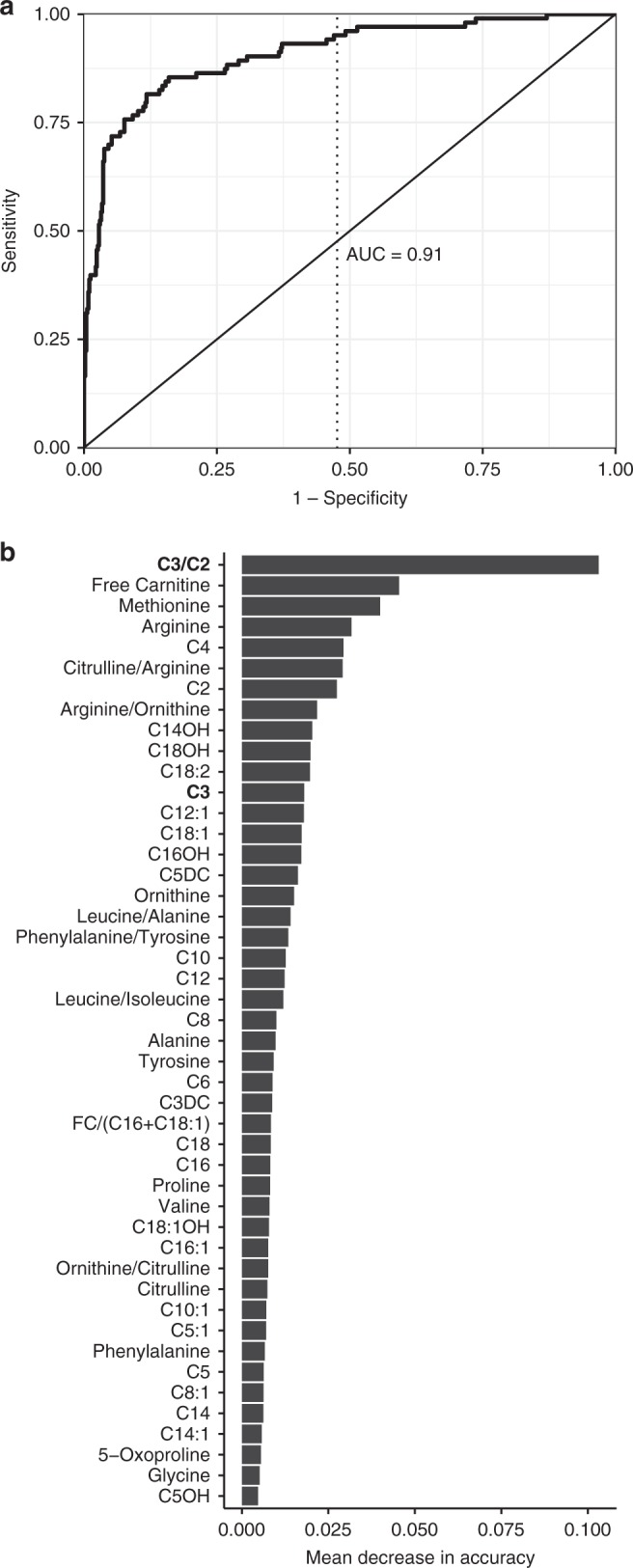
**Newborn metabolic pattern analysis with Random Forest (RF).** (**a**) Receiver operating characteristic (ROC) curve analysis for newborns with and without a confirmed methylmalonic acidemia (MMA) diagnosis using RF analysis of 46 MS/MS analytes. Without altering the 96.1% sensitivity of MMA screening based on the primary newborn screening (NBS) analyte C3 and C3/C2, RF reduced the number of MMA false-positive cases to 49% (vertical dotted line), which increased the positive predictive value (PPV) from 16.5 to 28.9%. (**b**) The mean decrease in accuracy (MDA) was used to measure the contribution of individual metabolic analytes in the RF model. The relative importance of analytes for MMA metabolic pattern recognition is ranked from top to bottom with primary markers C3 and C3/C2 in bold. *AUC* area under the curve.

We trained a machine learning classifier based on Random Forest (RF)^21^ that utilized 46 MS/MS analytes to distinguish true and false-positive MMA cases. Without changing the 96.1% sensitivity of MMA screening (99 of 103 true positives detected, 4 false negatives), RF reduced the number of MMA false positives from 502 to 244 (49%) and increased the positive predictive value (PPV) from 16.5 to 28.9% (Fig. 2a). The MDA index was used to identify the individual contribution of specific MS/MS analytes and covariates in our RF model. Comparing the MDA index results between two RF models (Fig. 2b, Supplementary Figure 2) showed similar ranking for a number of metabolic analytes (e.g., C3/C2, free carnitine, methionine, C4, arginine). Of the four covariates tested, newborn birth weight was the highest ranked covariate. RF analysis of two MMA phenotypic subgroups showed significant differences in analyte ranking for patients with mutase deficiency (e.g., C3/C2, C3, C12:1, C18OH) compared with patients with a cobalamin disorder (e.g., methionine, C3/C2, C18:2) (Supplementary Figure 3). Of the 95 MMA patients, RF misclassifed only 15 mut^0/-^ patients as CblC, D or F (or vice versa) with an error rate of 16%. Finally, of the 60 MMA cases sequenced, RF confirmed all 30 MMA cases as true positives and reduced the number of MMA false positives from 30 to 15 (Supplementary Table 1).

### RUSPseq quality control (QC) and data analysis

**Fig. 3:**
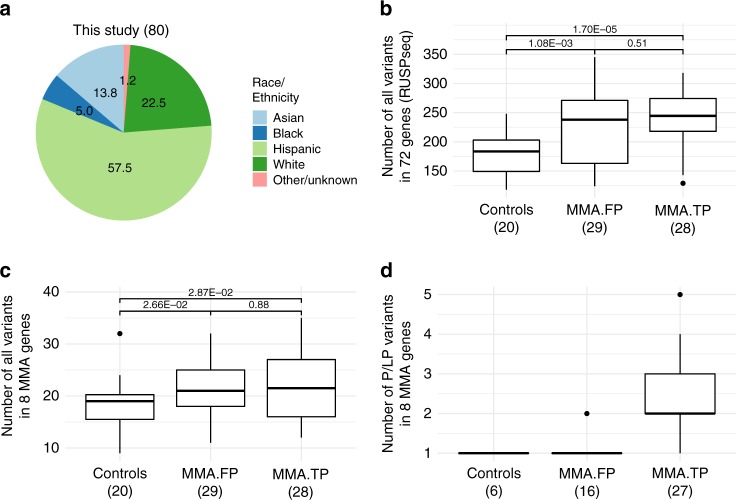
**Study population and sequence data analysis.** (**a**) Distribution of race/ethnicity among newborns sequenced in the three sample groups. (**b**) RUSPseq detected a relative larger number of DNA variants in methylmalonic acidemia (MMA) screen positives compared with gestational age (GA)/gender matched controls in all 72 genes, and (**c**) in the eight MMA-related genes, respectively. The sample size in each group is indicated at the bottom. (**d**) Pathogenic (P) and likely pathogenic (LP) variants in eight MMA genes were identified in each sample based on American College of Medical Genetics and Genomics (ACMG) standards and guidelines for interpretation of sequence variants.^28^ For each group, the number of samples with P/LP variants is shown at the bottom. Two P/LP variants were detected in one or more MMA genes in 24 MMA patients, while 2 additional patients had only one P/LP variant, and 1 patient had one P/LP variant and a variant of unknown significance (VUS). No such variant combinations (e.g., two P/LP, or one P/LP and one VUS) were detected in healthy controls, while 2 MMA false positives had two variants in a MMA gene located in *cis* on the same chromosome.

Here, we developed a recently validated multiplex NGS technology^16^ for sequencing of 72 genes for inborn metabolic disorders (RUSPseq) from DBS, which increased the number of primers pooled in a single tube by more than 20-fold. The 72 genes were curated based on evidence for association with RUSP metabolic conditions (Supplementary Table 2). Assay performance was assessed using our algorithms for monitoring sequence read coverage on four levels: sequence runs, samples, amplicons, and sequence base pairs. The first QC metric (sequence runs), defined as the percentage of all amplicon bases in 72 genes (362 kb) covered at a specified read depth, was used to compare the performance of different MiSeq runs (Supplementary Figure 4a). The second QC metric (sample coverage), defined as the number of reads per sample, was used for detecting samples that failed in the multiplex polymerase chain reaction (PCR). This metric identified sample G1 in run 2 with inadequate coverage (Supplementary Figure 4b). The third QC metric (amplicon coverage) was used to identify samples with partially failed amplification, such as individual amplicons that may have been insufficiently covered despite an overall normal read count for that sample. For each sample, we obtained the mean amplicon coverage and calculated the fraction of amplicons covered by 20% (0.2× mean) of the mean amplicon coverage. A threshold of 2 SDs below the mean of all samples was used to flag samples for review. This metric identified two samples (G1 and D1) with poor uniformity (Supplementary Figure 4c). Lastly, the fourth QC metric (base pairs) assessed base coverage for each sample, reasoning that if base coverage was sufficiently high, even samples with lower amplicon uniformity could be analyzed further. Sample G1 flagged in the prior QC steps had a low base coverage, while sample D1 passed this threshold and yielded interpretable results in sequence analysis. In summary, of the 77 samples that progressed to analysis, >90% of the target bases within the region of interest were covered at ≥20 reads (Supplementary Figure 4d), providing a high confidence for single-nucleotide variant (SNV) calling. Sequence analysis detected a larger number of variants in the 72 genes in the MMA screen-positive cases compared with controls (Fig. 3), which could be due to the high number of Hispanic newborns (72% in MMA.FP, 64% in MMA.TP) compared with controls (30%). In each of the 77 samples, variants in eight MMA genes (*MUT*, *MMAA*, *MMAB*, *MMACHC*, *MMADHC*, *LMBRD1*, *MCEE*, *ACSF3)* were manually classified based on ACMG guidelines,^28^ identifying a total of 73 unique pathogenic or likely pathogenic (P/LP) variants, of which 24 variants were listed in ClinVar.^26^ Twenty-five MMA patients were found with two P/LP variants or one P/LP and a variant of unknown significance in a MMA gene. Two MMA false positives (H10, E10) were identified with two variants in a MMA gene and read phasing showed that these variants were located in *cis* (Supplementary Figure 6**)**. None of the controls carried two DNA variants in a MMA gene.

## Discussion

Although MS/MS-based screening now identifies most newborns with MMA, it also creates a high number of false positives at a ratio of 5 infants without the disorder to 1 infant with the disorder. We identified statistically significant differences for several MS/MS analytes (Supplementary Figure 1), and explored if these analytes could be used in addition to the primary MMA analytes C3 and C3/C2 to improve the prediction of MMA true and false positives. Our approach using Random Forest (RF) machine learning reduced the number of false-positive MMA cases by 51% (from 502 to 244) without changing the 96.1% sensitivity of screening. C3/C2, C4, methionine, arginine, and the citrulline/arginine ratio were among the high-ranking analytes in the RF model (Fig. 2). In a separate analysis, postanalytical interpretive tools from Collaborative Laboratory Integrated Reports (CLIR)^29,31^ were applied to MS/MS screening data of our 605 MMA screen-positive cases. While CLIR tools initially had a reduced sensitivity for MMA true-positive cases, CLIR achieved a performance comparable with RF after including all 46 NBS analytes (unpublished results, Piero Rinaldo, personal communication). These results suggest that second-tier postanalytical performance is maximized by utilizing information on the entire MS/MS metabolic profile measured at birth. This novel approach may be used to reduce false-positive outcomes in other RUSP metabolic disorders. Furthermore, RF can also be used to study different MMA phenotypic subgroups. NBS using MS/MS does not currently distinguish between a complete or partial deficiency of methylmalonyl-CoA mutase (mut^0/-^) and impaired cobalamin metabolism (CblC, D or F) as a cause of MMA. A comparison of these MMA subgroups using RF analysis of 46 MS/MS analytes identified methionine as the highest-ranking analyte for separating CblC, D, or F patients from MMA false positives (Supplementary Figure 3). We estimated an error rate of 16% for RF to separate mut^0/-^ from CblC, D or F patients using MS/MS data.

Analysis of newborn race/ethnicity profiles reported by the California NBS program revealed that MMA screen-positive cases were more likely of Hispanic ethnicity, with both true and false positives showing similar race/ethnicity profiles (Fig. 1). While the birth prevalence for specific disorders is known to vary among different racial/ethnic groupings,^30^ identifying a higher number of MMA false-positive cases with Hispanic ethnicity was surprising. MMA screen-positive cases are detected in NBS based on elevated C3 or C3/C2 levels. Our first hypothesis was that Hispanic newborns may have a naturally higher level of C3 or C3/C2, which could directly lead to more MMA false positives in this ethnic group compared with non-Hispanic newborns. Our second hypothesis was that babies who are born prematurely may have higher levels of C3 or C3/C2. We found that preterms without or with MMA were otherwise very similar with respect to metabolite levels (Supplementary Figure 1), which in turn could make it harder for NBS to separate true from false positives. However, there is currently no support for substantially higher preterm birth rates for Hispanic newborns^32^ that would explain the high MMA false-positive rate in this ethnic group. Thus, more research is needed in a larger newborn population to test these hypotheses.

Second-tier DNA testing using DBS has been available for some RUSP diseases such as cystic fibrosis^4,33^ but it is not well established for metabolic disorders. Here we developed a multiplex sequencing assay from DBS for 72 genes for inborn metabolic disorders (RUSPseq) to enable comprehensive testing of these disorders in a time and cost-effective manner. We choose to pilot the assay for MMA screening because MMA is fraught with frequent false-positive results and DNA testing is often necessary to identify which of multiple genes is responsible, leading to diagnostic delays.^18^ RUSPseq was used to sequence 78 DBS samples with one sample (G1) flagged due to low read counts and 77 samples passing QC for variant analysis (Supplementary Figure 4). The 77 samples included 28 MMA patients, of which 25 patients (89%) were identified with a reportable molecular finding with two variants in a MMA disease gene (Fig. 3). This screening strategy was related to second-tier cystic fibrosis (CF) testing, which looks for two *CFTR* variants to minimize the referral of CF carriers for follow up.^33^ Of the 28 MMA patients, two patients (B2 and F4) had only a single P/LP variant, while one patient (F3) had only one VUS in a MMA gene (Supplementary Table 1). It is likely that these patients have yet unknown DNA changes in deep intronic and gene regulatory regions or in genes not targeted by this assay. Further expansion of RUSPseq to include additional genes that were not part of the current panel (e.g., *TCN2*, CD320, *ABCD4*, *HCFC1*, and *THAP11*) (refs. ^34–38^) may reveal MMA-related variants in these genes. While variants were found in all MMA-related genes, only *MUT*, *MMAA*, and *MMACHC* had two variants per gene in at least one patient. An overall agreement was seen between genes and phenotype (e.g., *MUT* and mut^0^, *MMACHC* and CblC, D or F). In two MMA false-positive cases (H10, E10) we identified two variants in a MMA gene located in *cis* on the same chromosome (Supplementary Figure 6). An area of future work is to reduce the time for variant interpretation, which ranged from <10 min to >2 h per variant. Similar to our curated CFTR database,^16^ RUSPseq data interpretation would greatly benefit from a database of curated metabolic genes. A major effort to establish and share such resources is underway in the Clinical Genome Resource (ClinGen) Inborn Errors of Metabolism Clinical Domain Working Group.^39^ A curated RUSP gene database could also shed light on challenges in variant interpretation in diverse multiethnic populations.^40^ Additionally, for new NGS assays to be adopted, NBS laboratories would need to perform thorough validation studies to show that the reliability of RUSPseq in a research setting will be maintained in the larger-scale clinical laboratory.

In this study, Random Forest–based analysis of the entire set of MS/MS screening data reduced the number of MMA false-positive newborns by more than half (51%) without altering clinical sensitivity. Applied to second-tier testing, RF analysis would immediately reduce the number of “false alarms” and help focus efforts on those newborns who require follow-up testing. RUSPseq multiplex gene sequencing from DBS provided a reportable molecular finding for 89% of the MMA patients, with preliminary evidence for no false-positive events. The remaining 11% false-negative patients (RF captured them correctly as true positives) would be routinely identified in biochemical testing using LC-MS/MS.^17^ Such combined second-tier approach from DBS would provide both genetic and metabolic information to the treating physician, and following clinical laboratory validation, could be implemented for rapid and inexpensive screening for MMA and other disorders in newborns.

## Electronic supplementary material

Supplementary Figure 1

Supplementary Figure 2

Supplementary Figure 3

Supplementary Figure 4

Supplementary Figure 5

Supplementary Figure 6

Supplementary Table 1

Supplementary Table 2

Supplementary Information
