# Supplementary material for: Combining newborn metabolic and DNA analysis for second-tier testing of methylmalonic acidemia
Source: Genet Med. 2018 Sep 13;21(4):896–903. doi: 10.1038/s41436-018-0272-5 (PMC6416784; doi:10.1038/s41436-018-0272-5)
Supplement: Supplementary file 1 — Supplementary Figure 1 [file 41436_2018_272_MOESM1_ESM.pdf]

	Controls−all	MMA.FP−all	MMA.TP−sub	Controls−preterm	MMA.FP−preterm	MMA.TP−sub−preterm	Controls−fullterm	MMA.FP−fullterm	MMA.TP−sub−fullterm
Phenylalanine/Tyrosine	7.4e−01	1.4e−05	7.8e−01	5.0e−01	8.9e−01	8.9e−01	4.1e−01	2.1e−07	4.1e−01
Citrulline	7.5e−01	4.2e−04	2.0e−01	2.7e−01	4.6e−02	1.7e−01	6.8e−01	2.0e−01	2.9e−01
Arginine/Ornithine	7.8e−01	3.2e−13	5.1e−01	7.4e−01	1.1e−01	4.8e−01	4.4e−01	2.8e−09	9.0e−01
Arginine	4.3e−01	3.0e−07	3.6e−02	6.3e−01	6.7e−01	4.6e−01	9.2e−01	6.9e−11	1.8e−01
Phenylalanine	3.8e−06	1.6e−04	5.3e−01	2.8e−01	2.7e−01	5.8e−01	3.7e−06	2.2e−01	8.4e−01
Tyrosine	7.2e−05	1.0e+00	1.7e−01	9.6e−01	3.5e−01	6.9e−01	6.2e−05	7.1e−04	4.9e−02
Alanine	9.6e−09	1.1e−01	4.5e−03	4.7e−01	9.8e−01	3.6e−01	2.8e−09	6.2e−02	2.4e−03
Ornithine	1.4e−01	3.1e−03	4.4e−02	4.0e−01	7.6e−01	4.2e−01	2.4e−01	1.8e−01	1.8e−01
Methionine	5.2e−01	1.5e−08	2.8e−11	6.1e−01	5.1e−01	1.9e−01	6.3e−02	2.5e−07	3.7e−09
Leucine/Alanine	1.4e−02	4.6e−10	1.4e−01	9.5e−01	3.9e−01	2.9e−01	5.1e−05	4.0e−05	3.6e−01
5−Oxoproline	1.1e−01	2.3e−01	1.1e−01	8.1e−01	1.6e−01	1.8e−01	1.6e−01	1.1e−01	4.2e−01
Glycine	4.0e−05	6.0e−01	1.2e−06	1.0e−01	8.7e−02	2.6e−01	7.1e−05	9.5e−01	3.1e−05
Ornithine/Citrulline	9.3e−02	1.3e−01	2.9e−03	1.8e−01	7.2e−01	9.6e−01	1.4e−01	6.4e−01	4.1e−03
Valine	2.1e−05	2.6e−06	1.6e−02	1.5e−01	3.7e−01	7.1e−01	1.2e−05	2.8e−02	1.5e−01
Citrulline/Arginine	2.9e−01	2.7e−07	2.3e−01	5.5e−01	1.7e−01	8.4e−01	3.6e−01	1.0e−04	1.3e−01
Leucine/Isoleucine	6.4e−03	4.0e−08	7.3e−02	3.0e−01	3.1e−01	9.6e−01	1.4e−02	1.1e−02	2.8e−01
C5DC	5.9e−01	2.7e−05	2.2e−01	9.3e−01	5.1e−01	6.6e−01	5.7e−01	5.9e−04	2.0e−01
C3DC	6.5e−01	1.7e−03	3.1e−01	9.5e−01	8.4e−01	6.6e−01	2.8e−01	1.8e−03	1.9e−01
C16	9.4e−01	2.5e−02	1.3e−02	4.9e−01	4.4e−01	8.6e−01	8.1e−01	5.7e−03	5.5e−04
C8:1	8.1e−01	3.9e−03	4.0e−02	6.4e−01	1.2e−01	6.0e−01	8.1e−01	2.4e−02	1.4e−03
C18:1	8.8e−01	1.9e−07	5.4e−04	4.5e−01	3.8e−01	4.0e−01	9.9e−01	4.2e−07	5.1e−06
C10	1.9e−02	1.4e−05	9.5e−01	2.8e−01	1.1e−01	8.8e−01	1.1e−01	2.8e−03	7.6e−01
C12:1	3.8e−02	7.4e−07	9.9e−01	1.6e−01	1.1e−01	6.0e−01	2.2e−01	4.3e−04	9.8e−01
C5:1	2.1e−04	5.9e−01	2.5e−01	7.5e−01	7.0e−01	1.2e−01	1.4e−03	4.4e−01	9.3e−01
C5OH	1.2e−03	5.7e−03	8.7e−02	9.4e−01	8.5e−01	2.8e−01	3.9e−03	7.2e−03	3.5e−01
**C3/C2**	4.5e−28	1.2e−10	5.6e−10	3.8e−05	9.8e−02	2.3e−01	3.5e−21	8.3e−09	6.6e−08
**C3**	5.3e−28	5.8e−03	4.9e−06	1.0e−05	4.4e−01	1.3e−01	7.9e−21	1.6e−02	1.2e−04
C2	3.5e−01	9.1e−14	1.2e−04	3.4e−01	9.0e−02	2.0e−01	3.2e−01	2.4e−10	1.1e−04
C18	4.6e−01	1.9e−02	6.3e−04	2.4e−01	2.1e−01	2.1e−01	5.4e−01	1.2e−01	1.2e−04
Free Carnitine	5.5e−02	4.6e−02	3.5e−01	5.7e−01	6.9e−02	1.3e−01	6.9e−02	1.4e−01	5.1e−01
C10:1	2.9e−01	1.3e−03	4.3e−01	5.8e−01	2.9e−02	1.5e−01	2.2e−01	7.3e−02	5.7e−01
C4	1.3e−03	8.8e−09	5.9e−03	1.0e+00	6.2e−02	2.5e−01	1.8e−03	5.9e−05	1.5e−02
C14	9.8e−04	8.8e−01	4.5e−03	3.5e−01	2.9e−01	3.9e−01	3.6e−03	2.4e−01	3.6e−03
C16:1	1.2e−04	5.6e−01	1.1e−01	1.3e−01	4.1e−01	6.2e−01	1.5e−03	4.3e−01	1.0e−01
FC/(C16+C18:1)	1.1e−01	4.1e−01	3.6e−01	5.5e−01	1.9e−01	2.9e−01	1.3e−01	7.8e−01	4.3e−01
Proline	1.9e−04	9.2e−02	4.2e−02	1.8e−01	9.5e−01	8.7e−01	3.1e−04	5.6e−01	6.5e−02
C18:2	5.2e−02	1.1e−15	8.9e−03	2.0e−01	2.5e−04	8.4e−01	1.4e−03	1.9e−05	1.1e−03
C18:1OH	3.3e−05	7.6e−03	3.7e−02	2.6e−01	1.5e−01	5.2e−01	1.6e−04	9.8e−02	8.0e−02
C14:1	6.0e−02	6.2e−02	2.0e−01	2.2e−01	2.3e−01	7.3e−01	2.7e−01	4.9e−01	1.6e−01
C8	2.1e−03	2.2e−01	3.3e−01	5.2e−01	1.1e−01	9.2e−01	1.9e−02	5.2e−01	3.6e−01
C18OH	1.3e−01	8.3e−04	2.2e−01	7.8e−01	1.2e−01	8.4e−01	1.7e−01	1.1e−02	1.5e−01
C6	2.0e−03	1.6e−01	3.3e−01	3.3e−01	2.6e−01	9.2e−01	1.2e−02	3.4e−02	4.2e−01
C5	1.8e−16	2.8e−06	4.6e−01	3.6e−01	2.1e−01	8.7e−01	2.0e−16	5.5e−02	5.9e−01
C12	9.1e−02	3.2e−04	2.3e−01	5.2e−01	1.5e−01	7.8e−01	1.3e−01	1.5e−02	3.2e−01
C16OH	1.7e−04	1.4e−05	2.7e−01	4.0e−01	2.2e−01	8.7e−01	4.9e−04	4.9e−04	2.7e−01
C14OH	2.6e−05	6.9e−06	1.8e−01	4.7e−01	3.3e−01	8.7e−01	6.7e−05	1.2e−04	3.1e−01
