# Supplementary figures and images for: Combining newborn metabolic and DNA analysis for second-tier testing of methylmalonic acidemia
Source: Genet Med. 2018 Sep 13;21(4):896–903. doi: 10.1038/s41436-018-0272-5 (PMC6416784; doi:10.1038/s41436-018-0272-5)

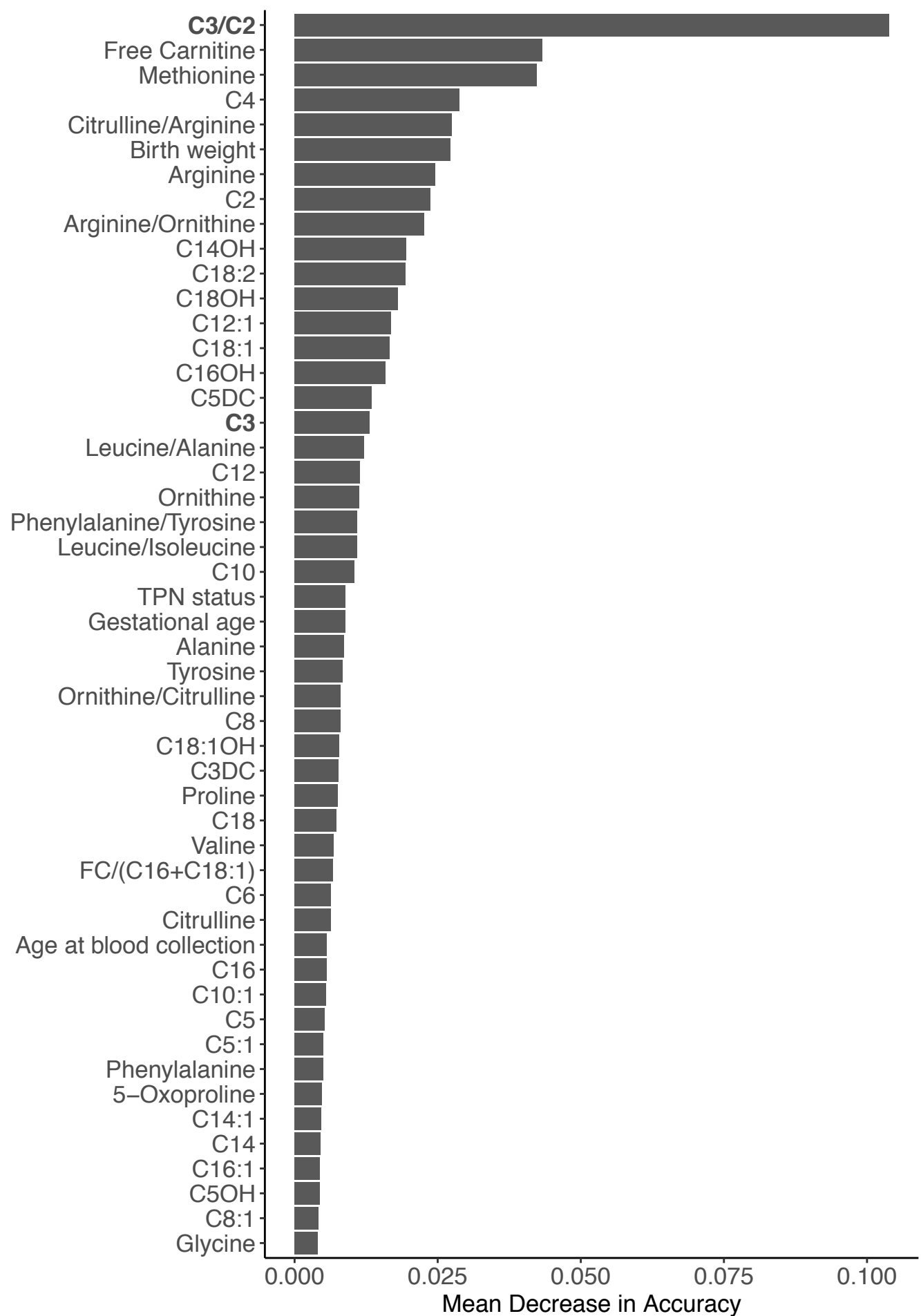

Supplement: Supplementary file 2 — Supplementary Figure 2 [file 41436_2018_272_MOESM2_ESM.pdf]

**A**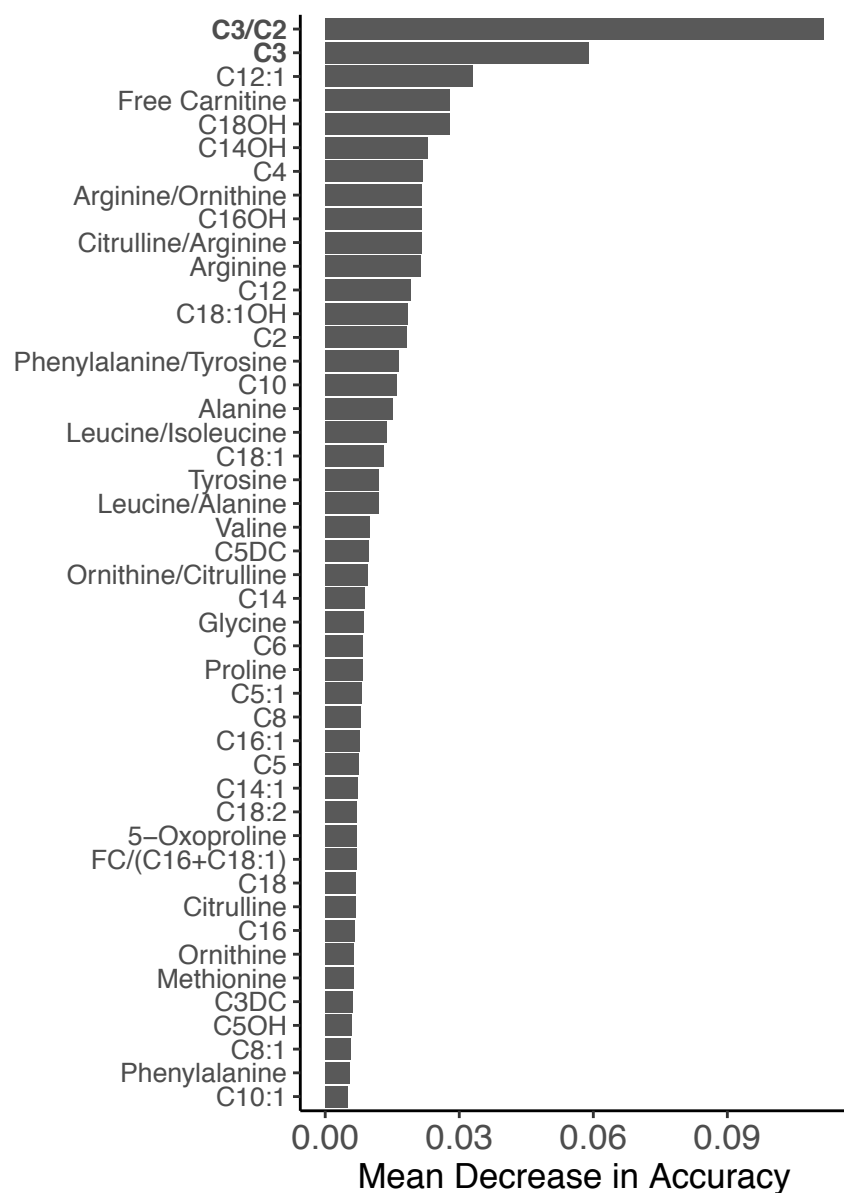**B**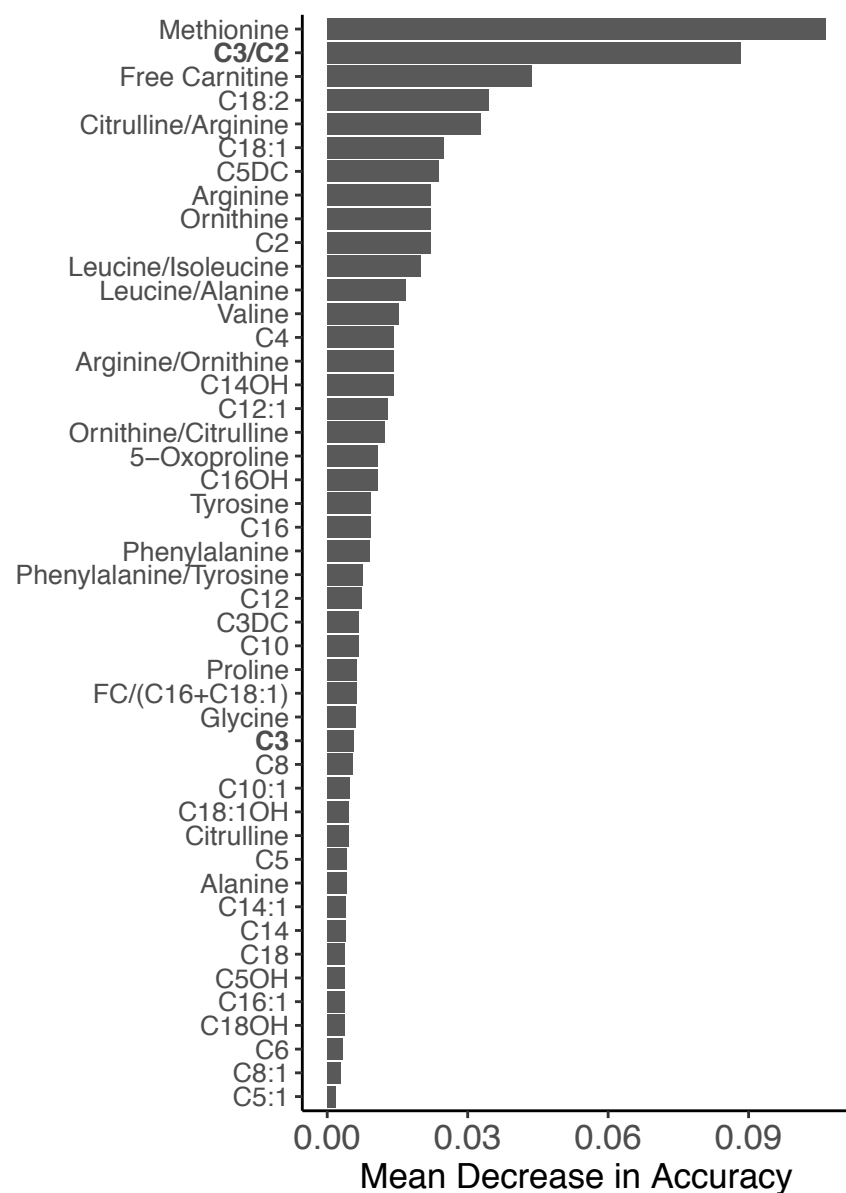**C**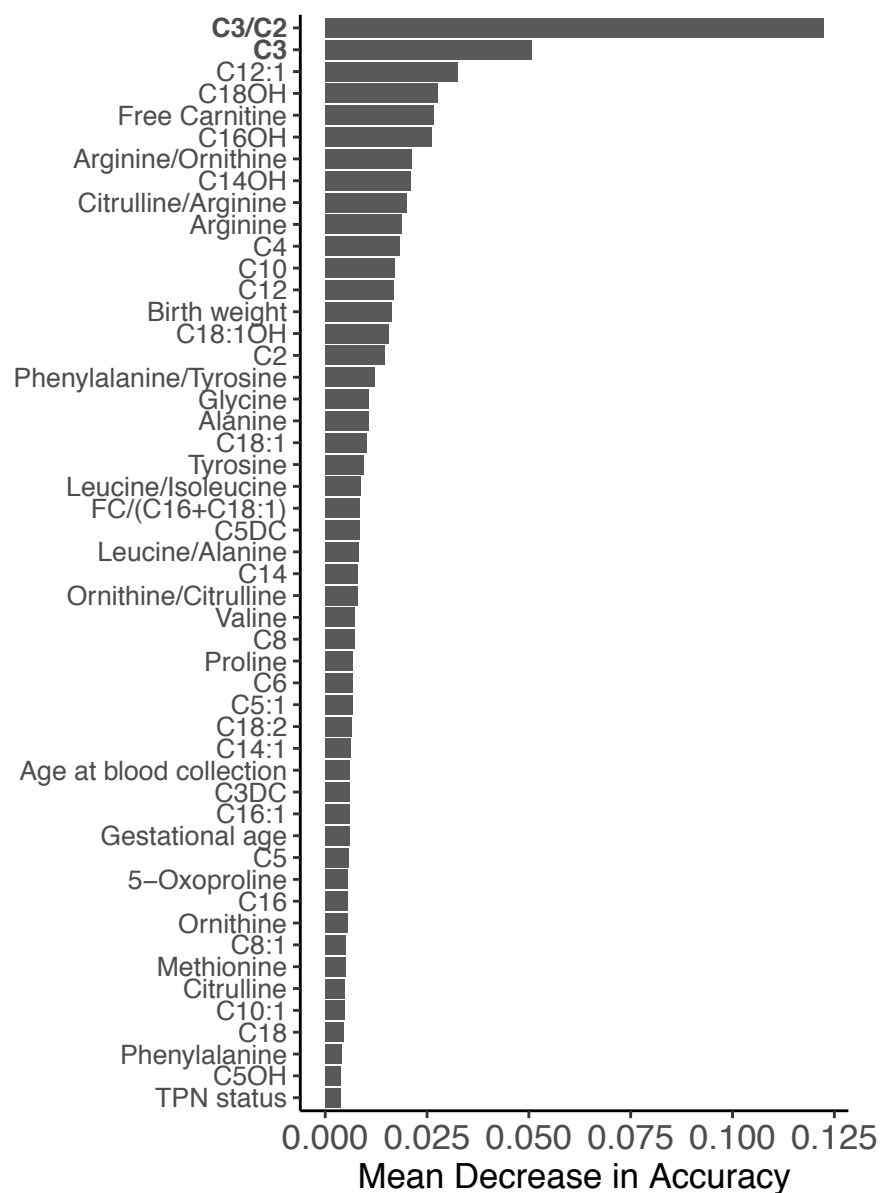**D**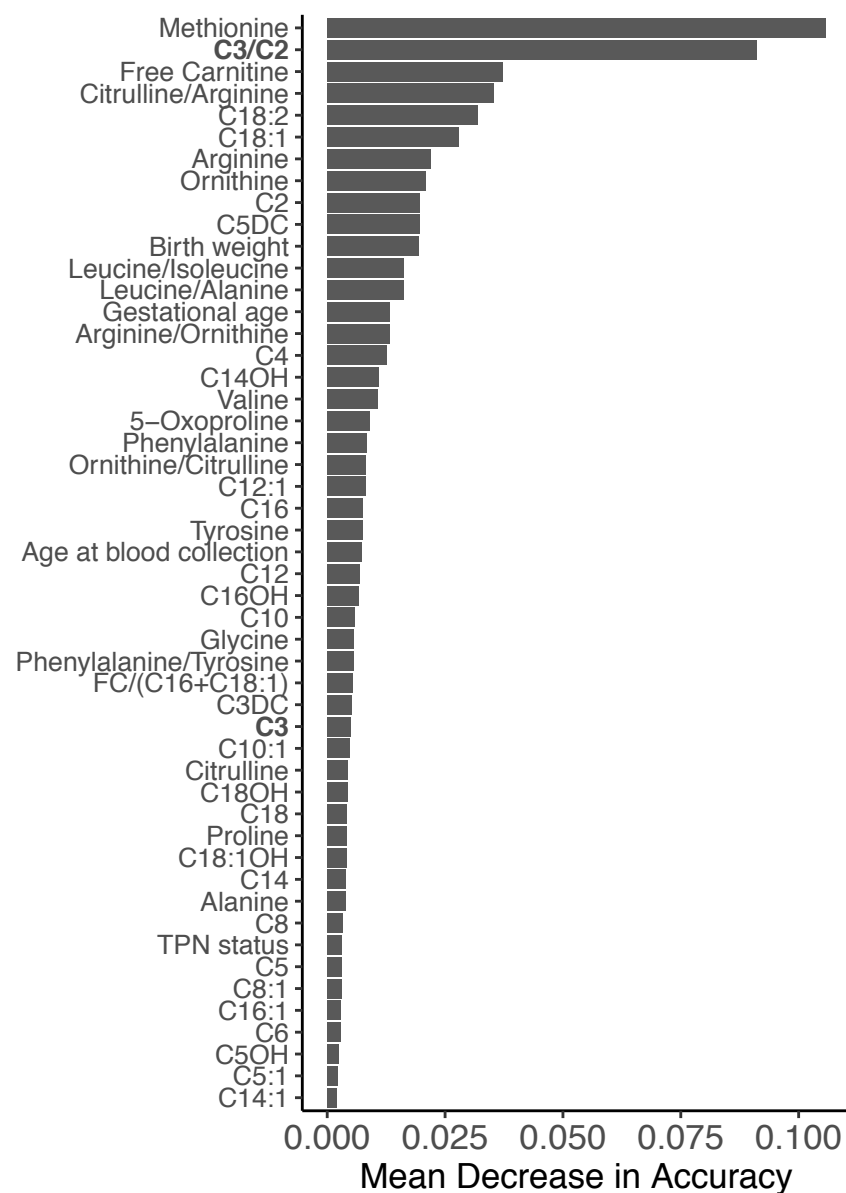

Supplement: Supplementary file 3 — Supplementary Figure 3 [file 41436_2018_272_MOESM3_ESM.pdf]

**A**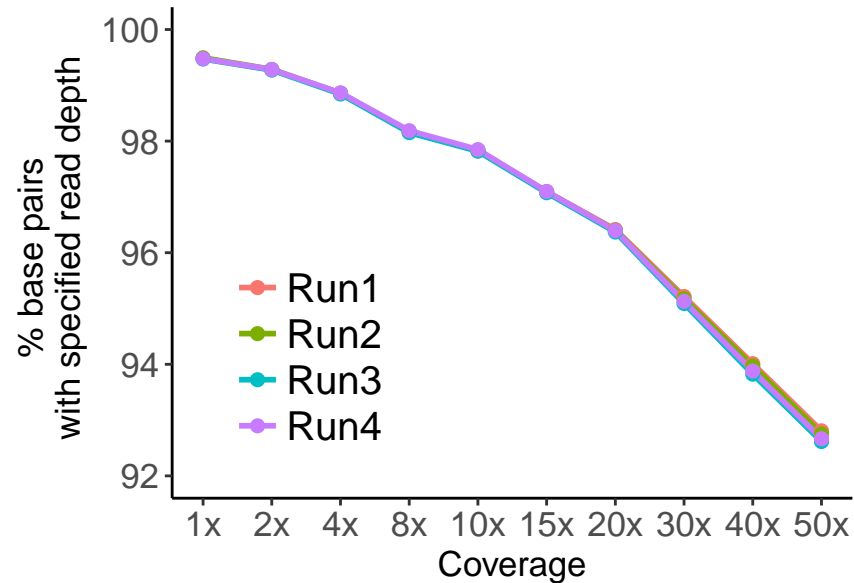**B**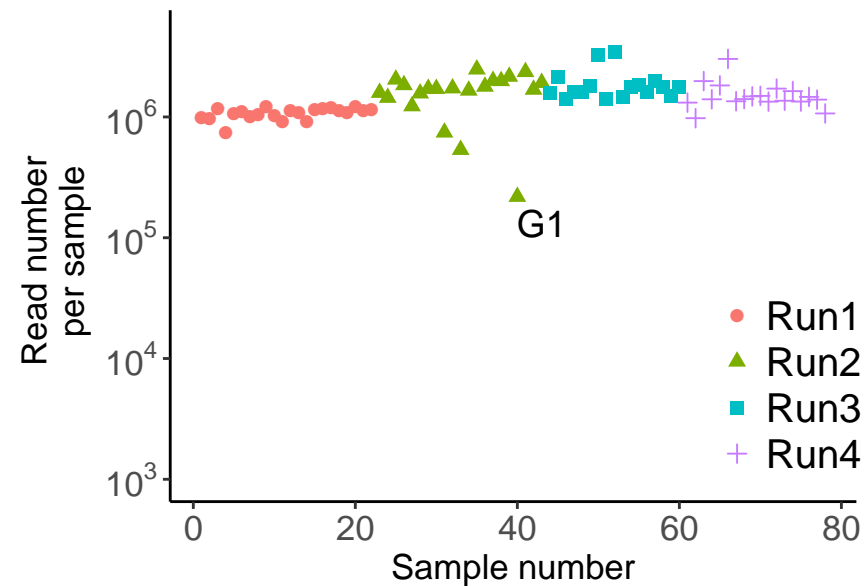**C**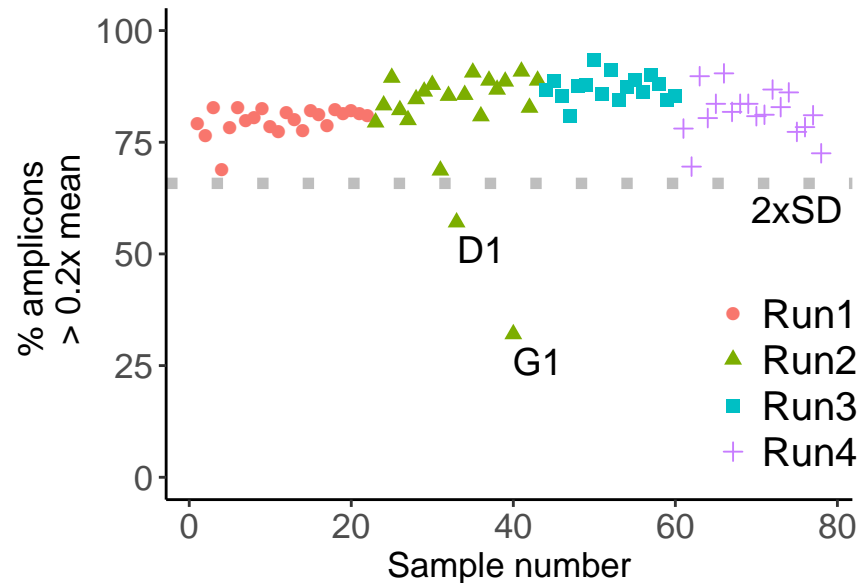**D**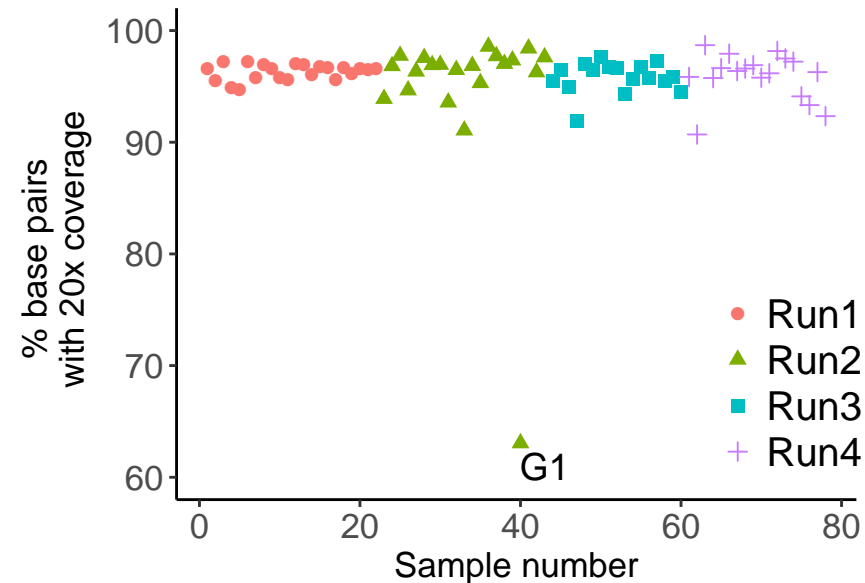

Supplement: Supplementary file 4 — Supplementary Figure 4 [file 41436_2018_272_MOESM4_ESM.pdf]

**A**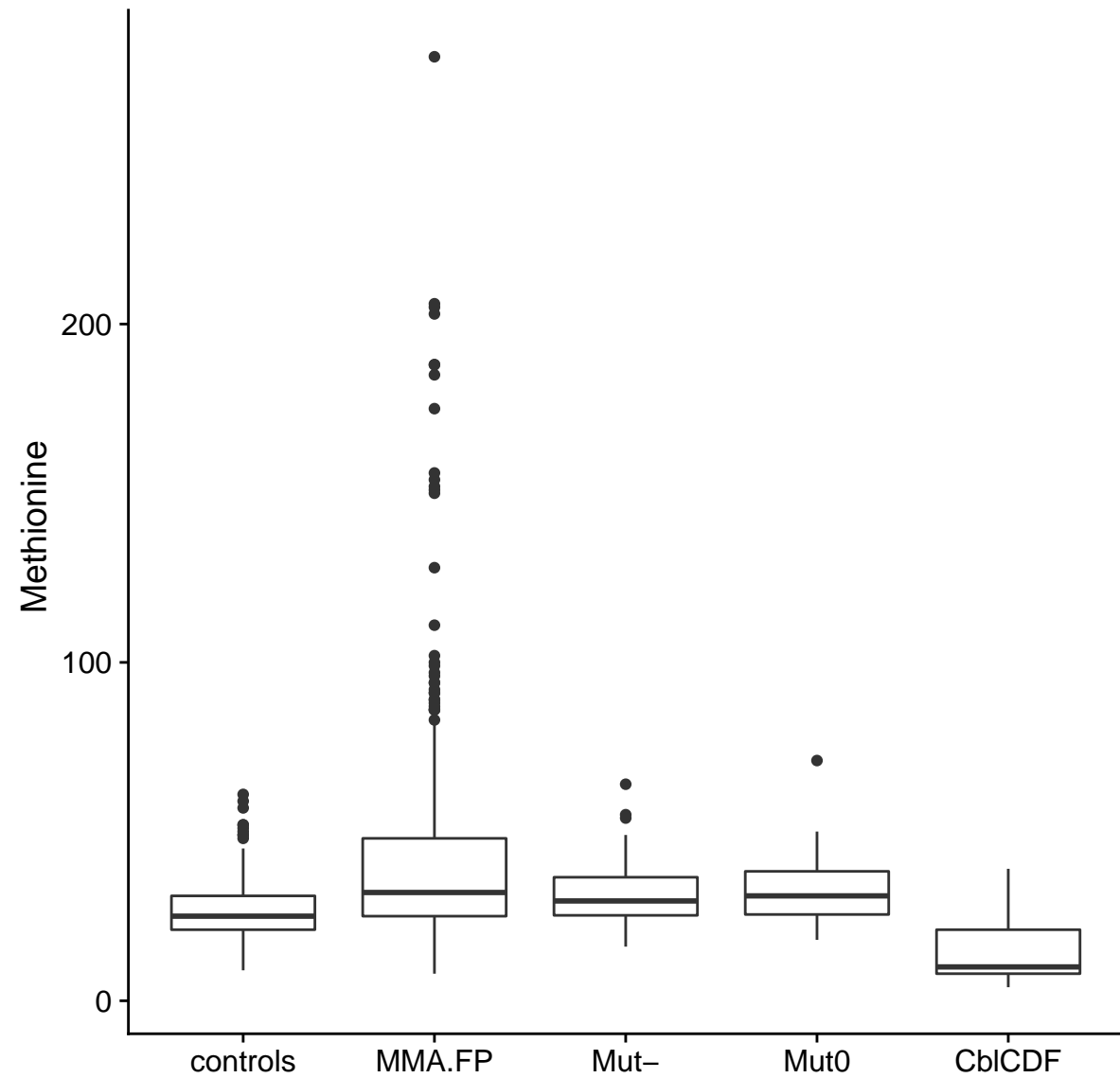**B**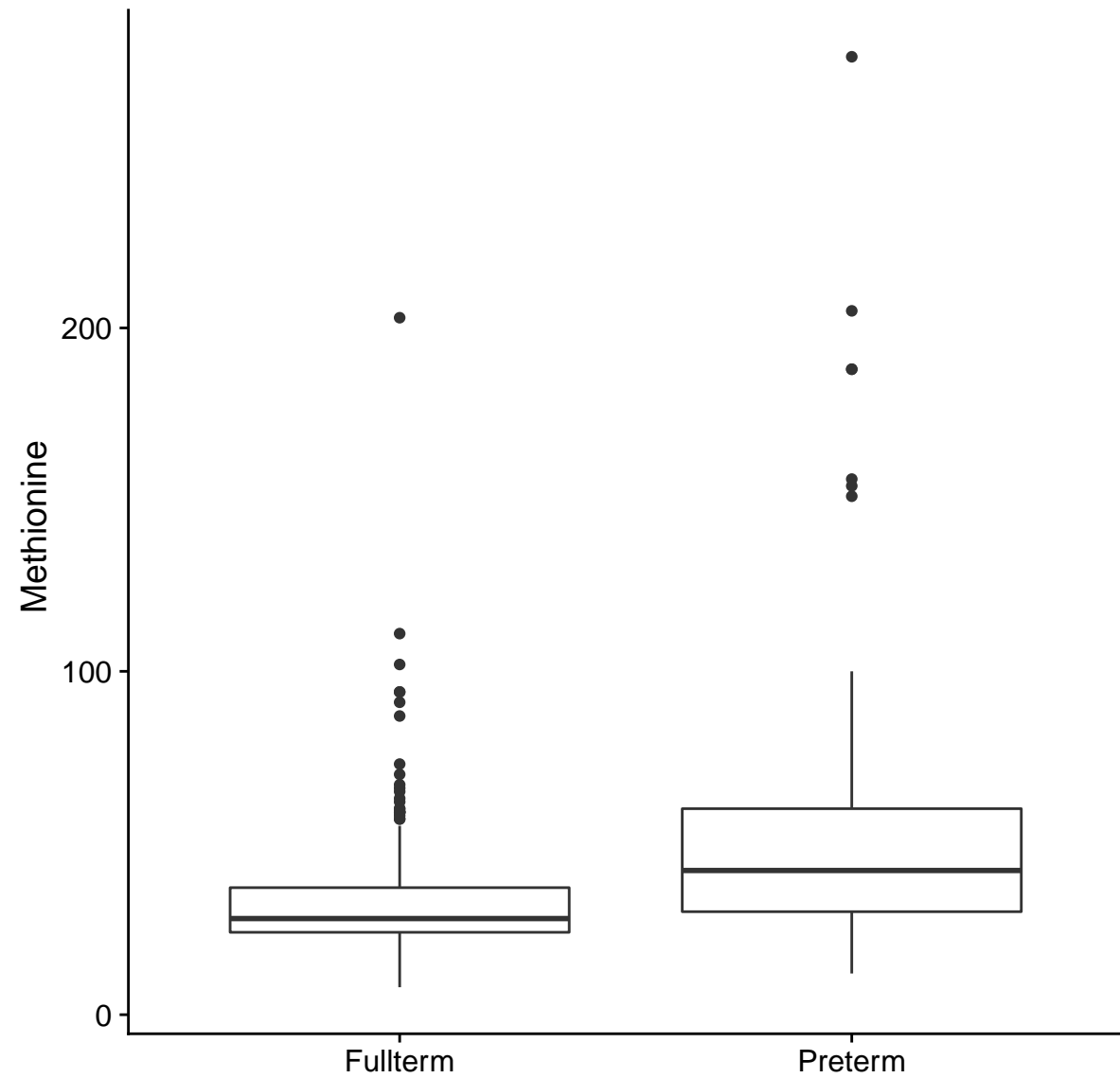

Supplement: Supplementary file 5 — Supplementary Figure 5 [file 41436_2018_272_MOESM5_ESM.pdf]
