# Supplementary material for: Combining newborn metabolic and DNA analysis for second-tier testing of methylmalonic acidemia
Source: Genet Med. 2018 Sep 13;21(4):896–903. doi: 10.1038/s41436-018-0272-5 (PMC6416784; doi:10.1038/s41436-018-0272-5)

Supplementary Figure 6: Phasing sequence variants in five samples.

(**a**) Sample C3 (patient): IGV screenshot shows the two MMACHC variants c.G608A:p.W203X (red arrow) and c.T578C:p.L193P (green arrow) located in trans on different reads.

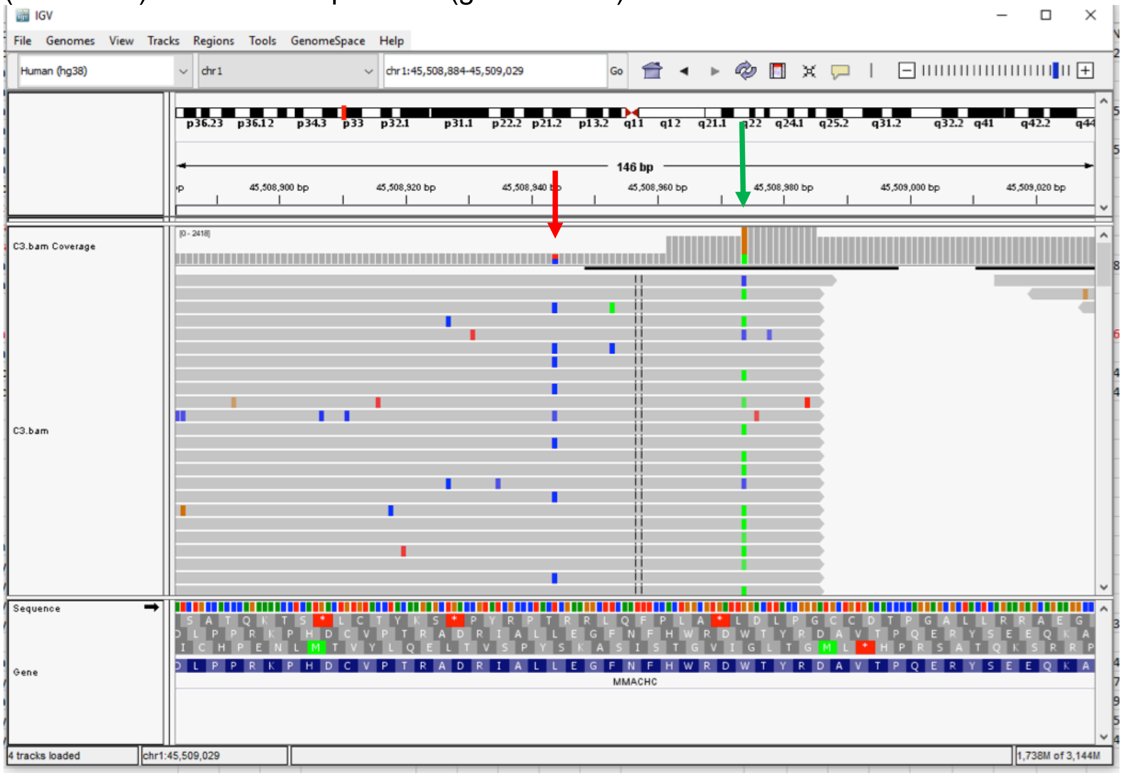

(**b**) Sample H10 (MMA.FP): IGV screenshot shows the two MUT variants c.1818delA:p.K606fs (red arrow) and c.G1810A:p.V604I (green arrow) located in cis on the same sequence reads.

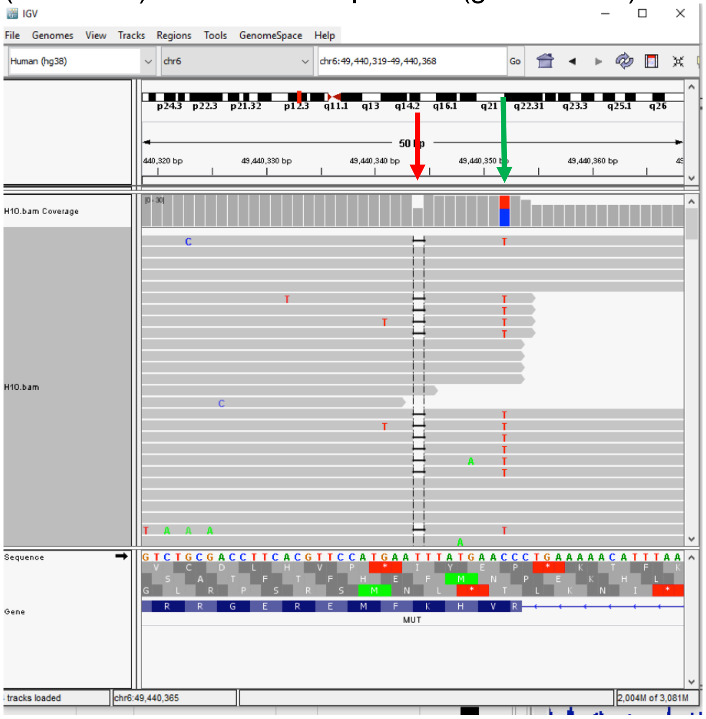

(**c**) Sample E10 (MMA.FP): IGV screenshot shows LMBRD1 variants c.C1321T:p.Q441X (red arrow) and c.C1242T:p.C414C (green arrow) located in cis on same sequence reads.

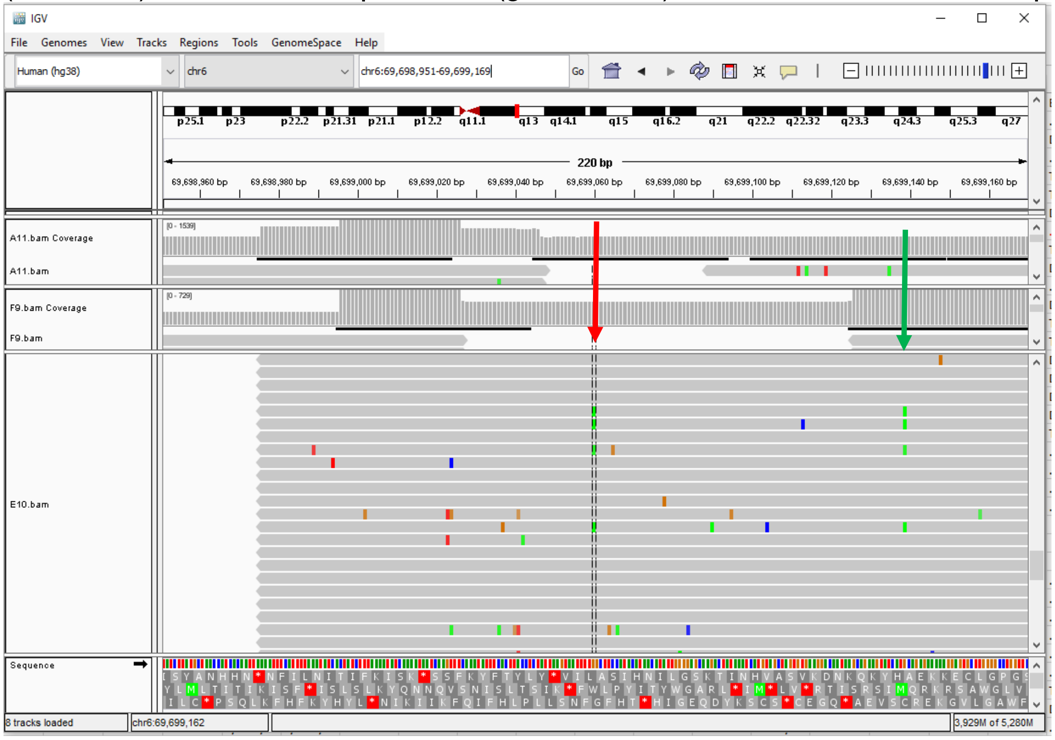

(**d**) Sample B8 (MMA.FP): IGV screenshot shows the two MLYCD variants c.799-2A>T (red arrow) and c.C886T:p.Q296X (green arrow) located in cis on the same sequence reads.

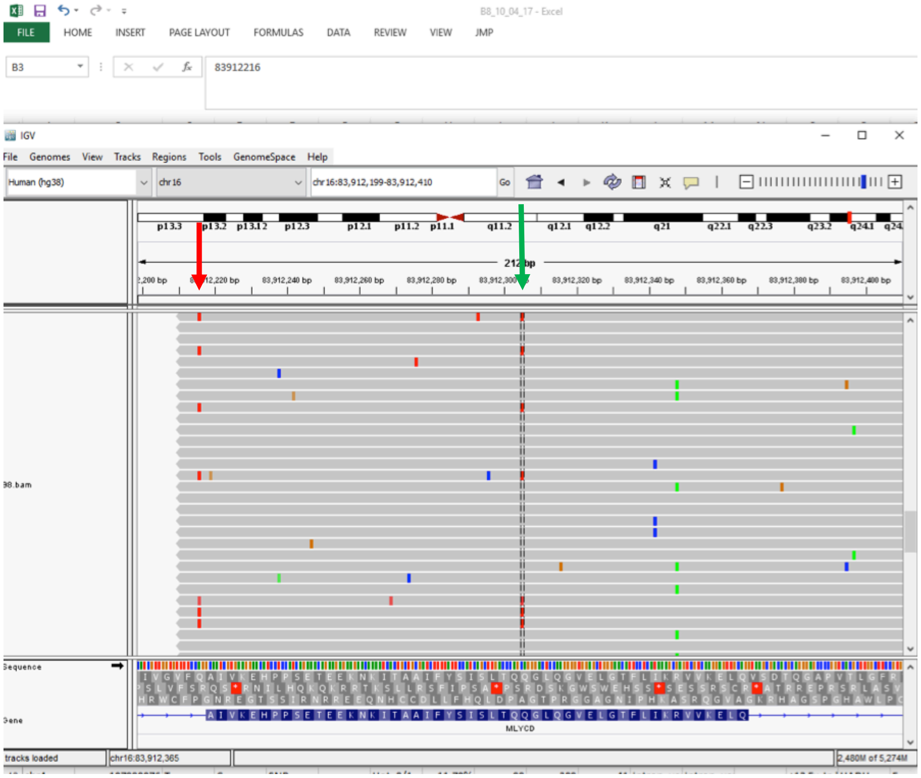

(**e**) Sample H6 (control sample): IGV screenshot shows the two PAH variants c.A286T:p.K96X (red arrow) and c.A204T:p.R68S (green arrow) located in cis on the same sequence reads.

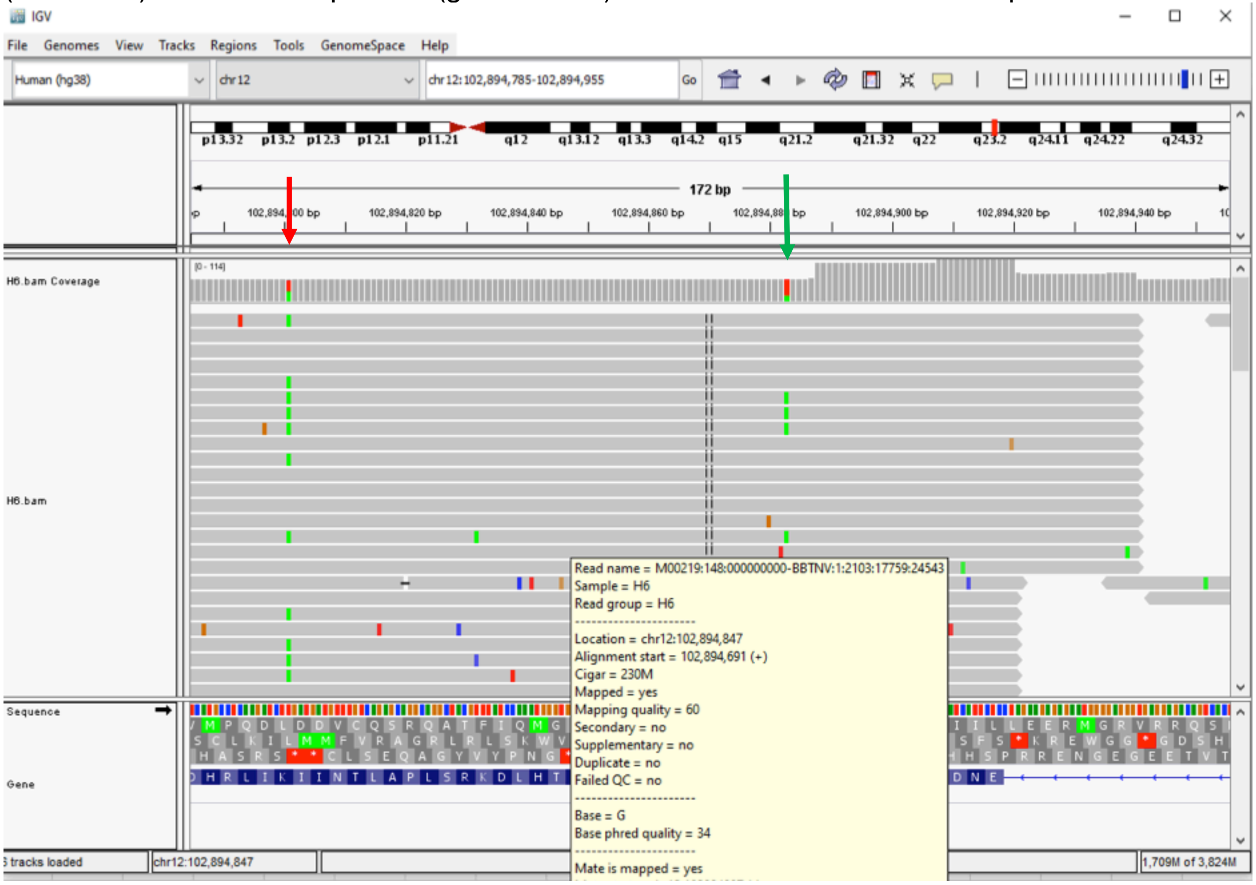

Supplement: Supplementary file 6 — Supplementary Figure 6 [file 41436_2018_272_MOESM6_ESM.pdf]
