# Supplementary material for: Combining newborn metabolic and DNA analysis for second-tier testing of methylmalonic acidemia
Source: Genet Med. 2018 Sep 13;21(4):896–903. doi: 10.1038/s41436-018-0272-5 (PMC6416784; doi:10.1038/s41436-018-0272-5)
Supplement: Supplementary file 8 — Supplementary Table 2 [file 41436_2018_272_MOESM8_ESM.docx]

Supplementary Table 2: Curation of 72 metabolic genes for RUSPseq.

|  | **Recommended Uniform Screening Panel (RUSP)*** | | | |  |  |  |  |
| --- | --- | --- | --- | --- | --- | --- | --- | --- |
| **RUSPseq** | **ACMG code** | **Conditions** | **Category** | **Condition type** | **Gene** | **NCBI Gene** | **NBS method** | **Primary analyte** |
| yes | PROP | Propionic Acidemia | core | Metab-OA | PCCA | 5095 | DBS, MS/MS | C3 |
|  |  |  |  |  | PCCB | 5096 |  |  |
| yes | MUT | Methylmalonic Acidemia (mut-0, mut-) | core | Metab-OA | MUT | 4594 | DBS, MS/MS | C3 |
| yes | Cbl-A,B | Methylmalonic Acidemia (Cobalamin disorders) | core | Metab-OA | MMAA | 166785 | DBS, MS/MS | C3 |
|  |  |  |  |  | MMAB | 326625 |  |  |
| yes | IVA | Isovaleric Acidemia | core | Metab-OA | IVD | 3712 | DBS, MS/MS | C5 |
| yes | 3-MCC | 3-Methylcrotonyl-CoA Carboxylase Deficiency | core | Metab-OA | MCCC1 | 56922 | DBS, MS/MS | C5-OH |
|  |  |  |  |  | MCCC2 | 64087 |  |  |
| yes | HMG | 3-Hydroxy-3-Methylglutaric Aciduria | core | Metab-OA | HMGCL | 3155 | DBS, MS/MS | C5-OH |
| yes | MCD | Holocarboxylase Synthase deficiency | core | Metab-OA | HLCS | 3141 | DBS, MS/MS | C5-OH |
|  |  |  |  |  | BTD | 686 |  |  |
| yes | BKT | Beta-Ketothiolase Deficiency | core | Metab-OA | ACAT1 | 38 | DBS, MS/MS | C5-OH |
| yes | GA1 | Glutaric Acidemia Type I | core | Metab-OA | GCDH | 2639 | DBS, MS/MS | C5-DC |
| yes | CUD | Carnitine Uptake Defect/Carnitine Transport Defect | core | Metab-FAO | SLC22A5 | 6584 | DBS, MS/MS | C0 |
| yes | MCAD | Medium-chain Acyl-CoA Dehydrogenase Deficiency | core | Metab-FAO | ACADM | 34 | DBS, MS/MS | C8 |
| yes | VLCAD | Very Long-chain Acyl-CoA Dehydrogenase Deficiency | core | Metab-FAO | ACADVL | 37 | DBS, MS/MS | C14:1 |
| yes | LCHAD | Long-chain L-3 Hydroxyacyl-CoA Dehydrogenase Deficiency | core | Metab-FAO | HADHA | 3030 | DBS, MS/MS | C16-OH |
|  |  |  |  |  | HADHB | 3032 |  |  |
| yes | TFP | Trifunctional Protein Deficiency | core | Metab-FAO | HADHA | 3030 | DBS, MS/MS | C16-OH |
|  |  |  |  |  | HADHB | 3032 |  |  |
| yes | ASA | Argininosuccinic Aciduria | core | Metab-AA | ASL | 435 | DBS, MS/MS | Citrulline |
| yes | CIT | Citrullinemia, Type I | core | Metab-AA | ASS1 | 445 | DBS, MS/MS | Citrulline |
| yes | MSUD | Maple Syrup Urine Disease | core | Metab-AA | BCKDHA | 593 | DBS, MS/MS | Leucine, Isoleucine |
|  |  |  |  |  | BCKDHB | 594 |  |  |
|  |  |  |  |  | DBT | 1629 |  |  |
|  |  |  |  |  | DLD | 1738 |  |  |
|  |  |  |  |  | BCKDK | 10295 |  |  |
|  |  |  |  |  | PPM1K | 152926 |  |  |
| yes | HCY | Homocystinuria | core | Metab-AA | CBS | 875 | DBS, MS/MS | Methionine |
|  |  |  |  |  | MTHFR | 4524 |  |  |
|  |  |  |  |  | MTR | 4548 |  |  |
|  |  |  |  |  | MTRR | 4552 |  |  |
| yes | PKU | Classical Phenylketonuria | core | Metab-AA | PAH | 5053 | DBS, MS/MS | Phenylalanine |
| yes | TYR-I | Tyrosinemia, Type I | core | Metab-AA | FAH | 2184 | DBS, MS/MS | Tyrosine, Succinylacetone |
| yes | BIOT | Biotinidase Deficiency | core | Metab-other | BTD | 686 | DBS, enzyme | Biotidinase |
| yes | CF | Cystic Fibrosis | core | Other-disorder | CFTR | 1080 | DBS, IRT | IRT |
| yes | GALT | Classical Galactosemia | core | Metab-other | GALT | 2592 | DBS, enzyme | Galactose, GALT activity |
| yes | Cbl-C,D | Methylmalonic acidemia with homocystinuria | secondary | Metab-OA | MMACHC | 25974 | DBS, MS/MS | C3 |
|  |  |  |  |  | MMADHC | 27249 |  |  |
| yes | MAL | Malonic acidemia | secondary | Metab-OA | MLYCD | 23417 | DBS, MS/MS | C3-DC |
| yes | IBG/IBD | Isobutyrylglycinuria | secondary | Metab-OA | ACAD8 | 27034 | DBS, MS/MS | C4 |
| yes | 2MBG | 2-Methylbutyrylglycinuria | secondary | Metab-OA | ACADSB | 36 | DBS, MS/MS | C5 |
| yes | 3MGA | 3-Methylglutaconic aciduria | secondary | Metab-OA | AUH | 549 | DBS, MS/MS | C5OH |
|  |  |  |  |  | OPA3 | 80207 |  |  |
|  |  |  |  |  | TAZ | 131118 |  |  |
| yes | 2M3HBA | 2-Methyl-3-hydroxybutyric aciduria | secondary | Metab-OA | HSD17B10 | 3028 | DBS, MS/MS | C5OH |
| yes | SCAD | Short-chain acyl-CoA dehydrogenase deficiency | secondary | Metab-FAO | ACADS | 35 | DBS, MS/MS | C4 |
| yes | M/SCHAD | Medium/short-chain L-3-hydroxyacyl-CoA dehydrogenase deficiency | secondary | Metab-FAO | HADH | 3033 | DBS, MS/MS | C4OH |
| yes | GA-II | Glutaric acidemia type II | secondary | Metab-FOA | ETFA | 2108 | DBS, MS/MS | C4 |
|  |  |  |  |  | ETFB | 2109 |  |  |
|  |  |  |  |  | ETFDH | 2110 |  |  |
| yes | MCAT | Medium-chain ketoacyl-CoA thiolase deficiency | secondary | Metab-FAO | HADHA | 3030 | DBS, MS/MS | C16OH |
|  |  |  |  |  | HADHB | 3032 |  |  |
| yes | CPT-IA | Carnitine palmitoyltransferase type I deficiency | secondary | Metab-FAO | CPT1A | 1374 | DBS, MS/MS | C0/C16+18 |
| yes | CPT-II | Carnitine palmitoyltransferase type II deficiency | secondary | Metab-FAO | CPT2 | 1376 | DBS, MS/MS | C16 |
| yes | CACT | Carnitine acylcarnitine translocase deficiency | secondary | Metab-FAO | SLC25A20 | 788 | DBS, MS/MS | C16 |
| yes | ARG | Argininemia | secondary | Metab-AA | ARG1 | 383 | DBS, MS/MS | Arginine |
| yes | CIT-II | Citrullinemia, type II | secondary | Metab-AA | SLC25A13 | 10165 | DBS, MS/MS | Citrulline |
| yes | MET | Hypermethioninemia | secondary | Metab-AA | MAT1A | 4143 | DBS, MS/MS | Methionine |
|  |  |  |  |  | AHCY | 191 |  |  |
|  |  |  |  |  | GNMT | 27232 |  |  |
| yes | H-PHE | Benign hyperphenylalaninemia | secondary | Metab-AA | PAH | 5053 | DBS, MS/MS | Phenylalanine |
| yes | BIOPT(BS) | Biopterin defect in cofactor biosynthesis | secondary | Metab-AA | GCH1 | 2643 | DBS, MS/MS | Phenylalanine |
|  |  |  |  |  | PTS | 5805 |  |  |
| yes | BIOPT(REG) | Biopterin defect in cofactor regeneration | secondary | Metab-AA | QDPR | 5860 | DBS, MS/MS | Phenylalanine |
|  |  |  |  |  | PCBD1 | 5092 |  |  |
| yes | TYR-II | Tyrosinemia, type II | secondary | Metab-AA | TAT | 6898 | DBS, MS/MS | Tyrosine |
| yes | TYR-III | Tyrosinemia, type III | secondary | Metab-AA | HPD | 3242 | DBS, MS/MS | Tyrosine |
| yes | GALE | Galactoepimerase deficiency | secondary | Other-disorder | GALE | 2582 | DBS, galactose | Galactose |
| yes | GALK | Galactokinase deficiency | secondary | Other-disorder | GALK1 | 2584 | DBS, galactose | Galactose |
|  | **Additional RUSPseq conditions (Not on the RUSP)*** | | | |  |  |  |  |
| yes | (OTC) | Ornithine transcarbamylase deficiency | - | Metab-AA | OTC | 5009 | DBS, MS/MS | Citrulline. Screened in CA since 2010. |
| yes | (CPS) | Carbamoyl-phosphate synthetase deficiency | - | Metab-AA | CPS1 | 1373 | - | No NBS. Included due to Hyperammonemia. |
| yes | (NAGSD) | N-acetylglutamate synthase deficiency | - | Metab-AA | NAGS | 162417 | - | No NBS. Included due to Hyperammonemia. |
| yes | (Cbl-F) | Methylmalonic aciduria and homocystinuria, Cbl-F type | - | Metab-OA | LMBRD1 | 55788 | DBS, MS/MS | Related to Cbl-A,B and Cbl-C,D. |
| yes | (CMAMMA) | Combined malonic and methylmalonic aciduria | - | Metab-OA | ACSF3 | 197322 | DBS, MS/MS | No NBS. Included due to MMA. |
| yes | (MCEE) | Methylmalonyl-CoA epimerase deficiency | - | Metab-OA | MCEE | 84693 | DBS, MS/MS | No NBS. Included due to MMA. |
| yes | (NKH) | Nonketotic hyperglycemia | - | Metab-AA | AMT | 275 | - | No NBS. Included due to DLD (PDH complex). |
|  |  |  |  |  | GLDC | 2731 | - |  |

*Reference: <https://www.hrsa.gov/advisory-committees/heritable-disorders/rusp/index.html>
