# Supplementary material for: Combining newborn metabolic and DNA analysis for second-tier testing of methylmalonic acidemia
Source: Genet Med. 2018 Sep 13;21(4):896–903. doi: 10.1038/s41436-018-0272-5 (PMC6416784; doi:10.1038/s41436-018-0272-5)
Supplement: Supplementary file 9 — Supplementary Information [file 41436_2018_272_MOESM9_ESM.docx]

**SUPPLEMENTAL INFORMATION**

RUSPseq primer design

We used our custom script integrating the primer design code from Primer 3^1^ to generate target-specific forward and reverse primers for 939 amplicons for 362,013 base pairs (bp) of all exons and 20 bp of flanking intronic sequence of 72 genes based on hg19/GRCh37 (**Supplementary Table 2**). Primer hybridization sites were selected to avoid common variants found in the National Center for Biotechnology Information (NCBI) single nucleotide polymorphism Database (dbSNP) build 137, June 2012 release. Primers were designed to have similar length (average 23 bp; range 21–27 bp), GC content, and amplicon size (average 412 bp, range 350-450 bp), matching the 2x250 bp paired-end sequencing chemistry on the MiSeq instrument (Illumina, San Diego, CA). Exons larger than 350 bp were covered by overlapping amplicons. Adapter sequences (24 bp) were included at the 5' end of each primer for post-capture amplification.

RUSPseq multiplex target capture

The 939 primer pairs were pooled in one tube for multiplex amplification of 72 genes. Establishing RUSPseq required careful primer design and primer pool rebalancing, that included increasing or lowering the concentration of specific primers, replacing of failed primers, sequencing and data analysis. Primer optimization minimized amplicon dropout and non-specific amplification and achieved a 99% target base coverage from <10 ng of DBS DNA. Multiplex PCR was performed in a Veriti 96-well thermal cycler (Applied Biosystem, Foster City, CA) using 4-6 μL of extracted DNA in a 20 μL final volume and the KAPA2G Fast Multiplex PCR Kit (Kapa Biosystems, Wilmington, MA) across the following thermal profile: 95°C for 3 minutes, 12 cycles of 95°C for 16 seconds, 69-52°C (-1.5°C per cycle) for 2 minutes, and 72°C for 45 seconds, followed by 10 cycles of 95°C for 16 seconds, 72°C for 20 seconds, and 72°C for 2 minutes. PCR cleanup was performed by adding 14 μL (0.7:1) of AMPure XP beads (Beckman Coulter, Brea, CA) and clean up according to the manufacturers manual, with a final elution in 14 μL elution buffer.

Sequence library construction and sequencing

We sequenced 78 samples in four MiSeq runs by multiplexing 17 to 22 samples per run. A no-template water control was included in each run. Sequencing library preparation was performed according to the manufacturer’s instructions (Illumina, San Diego, CA) using 5 μL of PCR product per sample. PCR was set up in 25 μL reactions, using common primers with sample specific indices and Illumina's P5 and P7 adapter sequences attached at the 5' end. Samples were barcoded with 8 bp dual indices according to Illumina's index sequencing protocol. KAPA2G Fast Multiplex PCR Kit (Kapa Biosystems, Wilmington, MA) was used to amplify DNA samples with the following cycling conditions: 98°C for 16 seconds, 13 cycles of 98°C for 16 seconds and 72°C for 20 seconds. Following DNA quantification for each sample, samples were pooled (approximately 200-400 μL total volume) and purified using AMPure XP beads (Beckman Coulter, Brea, CA) with a bead to sample ratio of 0.65:1 and eluted in 50 μL. We used 30 μL of the eluate for fragment size selection (440-720 bp) using the Pippin Prep system (Sage Science Inc., Beverly, MA), quantified the NGS library using Agilent Bioanalyzer (Agilent Technologies, Santa Clara, CA), and performed 2x250 bp PE sequencing on MiSeq (Illumina, San Diego, CA).

Sequencing data analysis

Image analysis and sample de-multiplexing was performed with the Illumina MiSeq Control Software version 2.4.1 and MiSeq Reporter version 2.5.1.3 (Illumina, San Diego, CA). The resulting processed fastq files were aligned to the GRCh38 human reference genome using the Burrows-Wheeler Aligner (BWA-MEM, version 0.7.13-r1126)^2^. Picard (version 2.8.1)^3^ was used to sort and convert files to BAM format. Quality control (QC) metrics were extracted for each sample from the BAM file, including total number of reads, percent reads that were properly paired and mapped to the reference genome, read depths for each amplicon, and read depth for individual base pairs within the target region (**Supplementary Figure 4**). The custom script for data analysis is available at <https://github.com/peng-gang/TGPipeline>.

**References:**

1. Rozen S, Skaletsky H. Primer3 on the WWW for general users and for biologist programmers. *Methods Mol Biol.* 2000;132:365-386.

2. Li H, Durbin R. Fast and accurate short read alignment with Burrows-Wheeler transform. *Bioinformatics.* 2009;25(14):1754-1760.

3. Li H, Handsaker B, Wysoker A, et al. The Sequence Alignment/Map format and SAMtools. *Bioinformatics.* 2009;25(16):2078-2079.

**Supplementary Figures**

**Supplementary Figure 1:** **Newborn metabolic patterns in different sample groups.** The row labels indicate 46 NBS metabolic analytes including 16 amino acids (top) and 30 acylcarnitines (bottom). The columns in the heat map are divided into three sample groups that include (from left to right) all 803 newborns, 198 preterm and 501 full-term newborns, respectively. In each of these three sample groups there are three columns comparing MMA patients to controls (1^st^ column) and MMA patients to MMA false-positives (2^nd^ column) with the p-values of the t-test shown for each comparison. The 3^rd^ column shows the p-values from ANOVA testing comparing three phenotypic subgroups of MMA patients (mut^-^, mut^0^, Cbl C,D,F). The primary MMA analyte C3 and ratio of C3/C2 were found significantly different between the patients, false-positives, and controls. Methionine was found to be significantly different between patients and false-positives, while it may also be an informative analyte for separating MMA phenotypic subgroups, with a higher value in mut^-/0^ as compared to Cbl. Metabolic analytes were not significantly different across the 198 premature newborns (middle sample group) indicating that preterm newborns are metabolically similar to each other.

**Supplementary Figure 2:** **The contribution of metabolic analytes in RF.** The Mean Decrease in Accuracy (MDA) was used to measure the contribution of individual metabolic analytes in the RF model (**Figure 2b**). In this analysis, four additional NBS covariates were incorporated in the RF model that includes birth weight in grams (Birth weight), total parenteral nutrition and hyperalimentation (TPN status), gestational age (GA) and the newborn age at blood collection in hours (Age at collection). While birth weight ranked high in the MDA analysis, the RF model predictions were highly similar to prediction solely based on NBS metabolic analytes.

**Supplementary Figure 3: Metabolic pattern analysis of MMA phenotypic subgroups.** NBS using MS/MS is not able to distinguish between a complete or partial deficiency of methylmalonyl-CoA mutase (mut^0/-^) and a cobalamin metabolism disorder (cblC, D, or F) as a cause of MMA. The Mean Decrease in Accuracy (MDA) was used to measure the contribution of 46 metabolic analytes in RF analysis of MMA phenotypic subgroups. In comparison to 502 false-positive cases for MMA, patients confirmed with mut^0/-^ (**a**) and cblC, D, or F (**b**) showed significant differences in MDA ranking of these analytes. After incorporating four additional covariates into the RF model, similar differences were found in MDA metabolite ranking for mut^0/-^ patients (**c**) and cblC, D, or F patients (**d**). Only small differences were observed in methionine levels between mut^0/-^ patients and MMA false-positives, while methionine was the highest-ranking analyte for separating cblC, D, or F patients from false-positives.

**Supplementary Figure 4: Quality control (QC) algorithm for DNA sequence data monitoring.** Read coverage was examined on four different levels: sequence runs, individual samples, amplicons, and base pairs. Samples that failed QC thresholds are indicated. (**a**) Average percent of bases covered at specified read depth in different sequence runs. For all samples in each sequence run, the average percentage of bp in 72 genes (362kb) with a specified read depth was calculated. (**b**) Total reads per sample for all 78 samples sequenced in four different MiSeq runs. One sample (G1) from Run 2 failed to amplify. (**c**) Uniformity of amplicon coverage. Shown is the percentage of amplicons with read coverage >0.2-fold the mean amplicon coverage for each sample. Two samples (D1 and G1) did not pass the empirically established threshold of (mean – 2xSD). (**d**) Shown is the percentage of bp with coverage >20 reads per bp for each sample. Only sample G1 had a lower bp coverage indicating that the remaining 77 samples could be analyzed further.

**Supplementary Figure 5:** **Methionine levels in different sample groups.** (**a**) The group of Cbl C,D, or F patients showed significantly lower levels of methionine, while mut^-/0^ patients had similar methionine levels compared to false positives (MMA.FP) and healthy controls. (**b**) Analysis of methionine levels in false positives showed statistical significant difference between full-term and preterm cases (p=7.09e-9), with relatively higher methionine levels in preterm false-positives.

**Supplementary Figure 6:** **Phasing sequence variants in five samples.** The haplotype phase of variants in a gene can be determined if they are located in close proximity within the same amplicon reads. (**a**) In patient C3, two MMACHC variants (c.T578C:p.L193P and c.G608A:p.W203X) were found on different reads for the same amplicon and are thus located in trans on different chromosomes. (**b**) In false-positive case H10, two MUT variants (c.G1810A:p.V604I and c.1818delA:p.K606fs) were found on the same amplicon reads and are thus located in cis in the same chromosome. (**c**) In false-positive case E10 two LMBRD1 variants c.C1321T:p.Q441X and c.C1242T:p.C414C were found on the same amplicon reads and are thus located in cis on same chromosome. (**d**) In false-positive case B8, two MLYCD variants (c.799-2A>T and c.C886T:p.Q296X) were found on the same amplicon reads and are thus located in cis in the same chromosome. (**e**) In control H6, two PAH variants (c.A286T:p.K96X and c.A204T:p.R68S) were found on the same amplicon reads and are thus located in cis in the same chromosome.

**Supplementary Tables**

**Supplementary Table 1: DNA sequence and metabolic data analysis in 80 newborns.** The 80 newborns included 30 confirmed MMA patients (mut^0^ or Cbl C,D,F), 30 MMA false-positives, and 20 controls. Random Forest-based analysis confirmed all 30 MMA patients as true positives and reduced the number of MMA false-positive cases from 30 to 15. Of 80 DBS, two samples (D3, C11) failed during DNA extractions and one sample (G1) was flagged with low read depth. The 77 sequenced samples included 28 MMA patients, of which 25 patients were identified with two variants in a MMA gene, while two patients (B2 and F4) had only one P/LP variant and one patient (F3) had no variant in the eight MMA genes analyzed. In the 29 MMA false-positive cases, we detected two samples (E10 and H10) with two variants in a MMA gene, which in both samples were found in cis on the same amplicon reads and are thus located on the same chromosome (**Supplementary Figure 6**). We did not detect two variants in a MMA gene in the 20 controls. Analysis of the other 64 genes in RUSPseq identified samples with two P/LP variants in *PAH*, *PCCA*, *MTHFR*, *MLYCD*, *HPD*, *ACADVL*, *FAH*, *CPS1*, *DBT*, and *NAGS*. In two samples (B8 and H6) the two P/LP were found on the same chromosome in *MLYCD* and *PAH*, respectively.

**Supplementary Table 2: Curation of 72 metabolic genes for RUSPseq.** The Recommended Uniform Screening Panel (RUSP) contains 60 conditions including 34 core conditions and 26 secondary conditions. RUSPseq contains 72 genes that include 64 genes associated with 46 different RUSP metabolic disorders and cystic fibrosis, and 8 genes associated with 7 metabolic disorders that are not currently on the RUSP, but which share metabolic-phenotypic similarities with RUSP metabolic disorders. Abbreviations: OA organic acid, FAO fatty acid oxidation, AA amino acid, DBS dried blood spot, MS/MS tandem mass spectrometry.
